# Pharmacological and metabolomic profiles of *Musa acuminata* wastes as a new potential source of anti-ulcerative colitis agents

**DOI:** 10.1038/s41598-022-14599-8

**Published:** 2022-06-22

**Authors:** Mona A. Mohammed, Bassant M. M. Ibrahim, Yasmin Abdel-Latif, Azza H. Hassan, Mohamed A. El Raey, Emad M. Hassan, Souad E. El-Gengaihi

**Affiliations:** 1 Medicinal and Aromatic Plants Research Department, Pharmaceutical and Drug Industries Research Institute, National Research Centre, Giza, 12622 Egypt; 2 Pharmacology Department, Medicine and Clinical Studies Research Institute, National Research Centre, Dokki, Giza, 12622 Egypt; 3 Medical Biochemistry Department, Medicine and Clinical Studies Research Institute, National Research Centre, Cairo, 12622 Egypt; 4 Faculty of Biotechnology, October University for Modern Sciences and Arts, 6th October, Giza, Egypt; 5 Pathology Department, Faculty of Veterinary Medicine, Cairo University, Giza, Egypt; 6 Department of Phytochemistry and Plant Systematics, Pharmaceutical and Drug Industries Research Institute, National Research Centre, Cairo, 12622 Egypt

**Keywords:** Biochemistry, Biological techniques, Chemical biology, Drug discovery, Immunology, Plant sciences, Biomarkers, Diseases, Gastroenterology, Health care, Medical research, Pathogenesis, Risk factors, Signs and symptoms

## Abstract

*Musa acuminata* (MA) is a popular fruit peels in the world. Non-food parts of the plant have been investigated for their antioxidant and anti-ulcerative colitis activity. Metabolomic approaches were found to be informative as a screening tool. It discovered different metabolites depending on statistical analysis. The antioxidant activity content was measured by colorimetric method. Seventy six investigated metabolites were observed. The identities of some of these markers were confirmed based on their MS^2^ fragmentation and NMR spectroscopy. These include: cinnamic acid and its dimer 2-hydroxy-4-(4-methoxyphenyl)-1H-phenalen-1-one beside; gallic acid and flavonoids; quercetin, quercetin-3-*O-β*-d-glucoside, luteolin-7-*O-β*-d-glucopyranoside. GC/MS analysis of MA peels essential oil led to identification of 37 compounds. The leaves, pseudostem and fruit peels extracts were tested for their safety and their anti-ulcerative colitis efficacy in rats. Rats were classified into: normal, positive, prednisolone reference group, MA extracts pretreated groups (250–500 mg/kg) for 2 weeks followed by induction of ulcerative colitis by per-rectal infusion of 8% acetic acid. Macroscopic and microscopic examinations were done. Inflammatory markers (ANCA, CRP and Ilβ6) were measured in sera. The butanol extracts showed good antioxidant and anti-inflammatory activities as they ameliorated macroscopic and microscopic signs of ulcerative colitis and lowered the inflammatory markers compared to untreated group. MA wastes can be a potential source of bioactive metabolites for industrial use and future employment as promising anti-ulcerative colitis food supplements.

## Introduction

Local body response to continuous tissue irritation by mechanical trauma or recurrent infection or chemical injury leads to inflammatory response in the form of swelling, redness and pain. Inflammation occurs as a defensive mechanism against spread of traumatic insult of irritating agent which leads to occurrence of inflammatory activity and subsequent threat to affected organ. Inflammatory diseases can affect the patient’s life quality adversely as it may lead to progression to more serious conditions that hinder normal activity as neuritis, osteoarthritis and digestive system inflammation that may progress to ulceration, which necessitates great care and continuous treatment with specific regimens^1,2^.

The most serious digestive system inflammatory diseases are those of bowel diseases including ulcerative colitis which affect a considerable range of population around the world. The rate of occurrence of ulcerative colitis (UC) is very high and is continuously increasing yearly. It affects patients at early adolescence and continues to progress throughout life^3^. One of the well-established characteristic of UC is chronic, colonic mucosal inflammation that is usually relapsing and is manifested by episodic attacks of severe abdominal pain that persist for a period of time and is associated with catarrhal or bloody diarrhea^4^.

Unfortunately as a whole current medication aren’t always to the expectations and have adverse effects on patient’s health, as treatment of mild or moderate UC is achieved by 5-aminosalicylates or sulfasalazine, while moderate or severe UC are treated by high-dose of oral or intravenous corticosteroid. However primary remission may not be accessed for all patients and consequently corticosteroid dependence or resistance may occur^5^, which may end up by total or partial colectomy^6^. That’s why there is need for introduction of supplementary herbal medicines in the regimen of treatment of UC provided that they don’t have evidenced side effects^1^. *Musa* spp. (bananas) is a good sources of carbohydrates, proteins, other vitamins and minerals. They contain different amino acids like threonine, tryptamine, tryptophan, as well as flavonoids, dopamine, beta-carotene and sterols^7^. Studying the biological activities of banana different parts was carried out in various studies; where stem were studied as antidiabetic supplements which improved the level of insulin and reduced blood glucose as well as glycosylated haemoglobin through modulating carbohydrate metabolizing enzymes activity^8,9^, also they have glycaemic effects oweing to their high content of sodium and potassium, fruit peels were studied as healing and anti-ulcerative agents, moreover methanolic as well as aqueous extracts of *Musa paradisiaca* (banana) were reported to produce wound healing in rats^7,10^, peel were studied for their immunomodulatory effects^11^. Additional evidence of the healing activity of *Musa paradisiaca* is that when ulcer was induced in experimental animals using non- steroidal anti-inflammatory drugs or steroid or histamine, oral administration of banana pulp powder showed marked antiulcerogenic effect^12^.

Phytochemical profile studying using a metabolomic approach of Leaves of bananas in previous studied using UPLC–QToF–MS technology showed that thirty-one compounds were identities of some of these markers based on their MS^2^ fragmentation. These include quercetin-*O*-rhamnoside-*O* hexoside, kaempferol-3-*O*-rutinoside, quercetin-*O-*hexoside, isorhamnetin-*O*-rutinoside and hexadecanoic acid.

Though accessions by soxhlet gave better yield (20.0–60.0%) than by sonication (18.4–23.0%),neither motherland nor methods of extraction had any significant role in the separation process^7^.

This research was carried out to investigate the chemical composition, and phytochemicals of some banana by-products; leaves, stem and fruit peels and study their potential protective effects against colonic inflammatory insult induced by acetic acid in rats, to mimic signs and symptoms of the serious devastating inflammatory bowel disease “ulcerative colitis” aiming at introducing a new functional anti-inflammatory food supplement.

## Material and methods

*Guideline ethics for plant usage in the phytochemical study* The present study complies with local and national guidelines as permission was obtained for collection of plant material.

Guideline ethics for experimental animal handling in the in vivo pharmacological study The study was done in accordance with the guide for care and use of laboratory animals Experiments were performed according to the National Regulations of Animal Welfare and the Institutional Animal Ethical Committee (IAEC) in Egypt, and is reported in accordance with Animal Research: Reporting of in vivo Experiments (ARRIVE) guidelines.

Ethical approval was obtained from National Research Centre ethics committee under number 16/138.

### Phytochemical study

#### Chemicals

ABTS, DPPH, Trolox and FCR were purchased from Sigma Aldrich (GmbH). All chemicals and solvents used in this study were supplied by Fisher Scientific UK (Bishop Meadow Road, Loughborough) with high analytical grade.

#### Plant materials, extraction, isolation and identification

*Musa acuminata* waste parts (leaves, pseudo-stem and fruit peels) were collected from a private farm at Anshase, Sharqia Governorate, Egypt and identified by Dr. Mohamed El Gebally, Former researcher of plant taxonomy, National Research Center and Senior of of plant taxonomy at Orman Botanical garden, Giza, Egypt. Herbarium specimens no S7570, S7571 and S5772, respectively were deposited at the herbarium of the National Research Centre, Dokki, Cairo, Egypt. The powdered MA wastes (0.5 kg) individually were exhaustively extracted three times with 5L of methanol at room temperature, then filtered and concentrated under reduced pressure at 45 °C using rotary evaporator to yield crude extracts 75.5 g (stem), 81.7 g (leaves) and 87.2 g (fruit peels). The three crude residues were suspended in 1L water, left overnight and then it was successively partitioned with 0.7 L of petroleum ether, chloroform three times, followed by 0.7 L ethyl acetate then 0.7 L n-butanol three times repeatedly.

Chromatographic procedure for isolation and identification of some phenolics compounds from three kg of powdered leaves were extracted with EtOH (80%) by soaking at room temperature. The combined alcoholic extracts were concentrated under reduced pressure at 45 °C, which yield 365 g of residue. The crude residue was suspended into water then overnight partitioned with petroleum ether, methylene chloride, ethyl acetate and n-butanol. The n-butanol fraction was chosen for further purification. The n-butanol fraction (g) was loaded on a column (3.5 cm × 150 cm) of Polyamide, and the column was stepwise eluted with water, 30, 50, 70, and 100% methanol at a flow rate of 50 mL/min to yield five sub fractions. 50% and 70% methanol sub-fraction was further purified using a sephadex LH-20 column chromatography and eluted with 50% methanol, to yield 6 compound. The jar of different elution solvent systems were used (BAW 4:2:1 or 30:10:10, acetic acid 15%, and Benzene: MeOH: Acetic Acid 45:8:4). After air-drying, the spots were visualized under UV light^13^. From 50% of alcohol gives five compound flavonoids and phenolics acids and one compound from 70% alcohol that identified by spectral tools data.

#### Appraising, detecting, identifying and characterizing secondary metabolites

##### GC/MS of essential oil fruit peels extract

The volatile oil of waste fresh fruit peels MA was extracted by water distillation method as Solvent-Assisted Flavour Evaporation (SAFE). The homogenate was continuously steam-distilled by diethyl ether (25 mL) extracted in 3 h in a Likens-Nickerson apparatus^14^. The resulted essential oil was separately dehydrated with anhydrous sodium sulphate and kept in deep freezer at − 20 °C until GC/MS analysis. The analysis was done in triplicate and the mean values of the oil content (%) were recorded. The components of essential oil in MA were identified by GC/MS analysis instrument stands at the Department of Medicinal and Aromatic Plants Research, National Research Center with the following specifications. Instrument: a TRACE GC Ultra Gas Chromatographs (THERMO Scientific Corp., USA), coupled with a THERMO mass spectrometer detector (ISQ Single Quadrupole Mass Spectrometer). The GC/MS system was equipped with a TG-WAX MS column (30 m × 0.25 mm i.d., 0.25 μm film thickness). Analyses were carried out using helium as a carrier gas at a flow rate of 1.0 mL min^−1^ and a split ratio of 1:10 using the following temperature program: 60 °C for 1 min; rising at 4.0 °C min^−1^ to 240 °C, and held for one minute. The injector and detector were held at 210 °C. Diluted samples (1:9 diethyl ether, v/v) of one μL of the mixtures were always injected. Mass spectra were obtained by (EI) at 70 eV, using a spectral range of *m/z* 40–450. Most of the compounds were identified using the analytical method: mass spectra (authentic chemicals, Wiley spectral library collection and NSIT library)^15^.

##### HPLC & LC/MSMS of secondary metabolites from MA wastes

(a) Quantitative of phenolic compounds from Butanol fractions of three parts of MA were measured using authentic fifteen standards. The HP 1100-HPLC system (Agilent Technologies, Palo Alto, CA, USA) with an auto-sampler (G1329B), quaternary pump and a diode array detector.

The measurements were integrated by chemstation chromatographic software interfaced to a personal computer. The analytical column was ZORBAX Eclipse XDB C18 column (15 cm × 4.6 mm I.D, 5 µm, USA). Operative conditions were: mobile phase A, 2% acetic acid; mobile phase B, acetonitrile; flow rate, 0.85 mL/min; fixed wavelength, 280 and 360 nm; injected quantity, 10 µL; elution program (%), A:B as followed: 0 min 90/10; 10 min 50/50; 15 min 20/80; 20 min 90/10; 25 min 90/10. Identification of phenolic compounds was performed by comparison with the retention times of standard substances.

(b) Instrument stands at the department of Metabolomics Groups, Institute of Plant Genetics of the Polish Academy of Sciences, Poznan, Poland, with the following specifications: first instrument; ion-trap Esquire 3000 mass spectrometer equipped with ESI was operated in negative ion mode with scan range was 15–3000 m/z and scan resolution was 13,000 m/z/s^16^. The second instrument; UPLC (the Acquity system, Waters, Milford, USA) coupled to Q-Exactive hybrid MS/MS quadrupole—Orbitrap mass spectrometer (Thermo, Bremen, Germany). The system of separation Chromatography was carried out using solvent (A) water acidified with 0.1% formic acid and (B) acetonitrile (solvent B). The flow of mobile phase 0.4 mL/min was adapt to the following sequences: 0–15 min 95:5, 15–22 min 50:50, 5 min for maintained the conditions 0.2:98, then system returned to the starting conditions and was re-equilibrated for 3 min. with column C18 (150 × 2.1 mm, 1.7 μm). Q-Exactive MS was operated upon following settings: the HESI ion source voltage (− 3 kV or 3 kV). The sheath gas (N_2_) flow 48 L/min, auxiliary gas flow 13 L/min, ion source capillary temperature 250 °C, auxiliary gas heater temperature 380 °C. The CID MS/MS experiments were performed using collision energy of 15 eV. Data recording and processing were performed using the Xcalibur 4.0 software with accuracy error threshold at 5 ppm and Imported data from raw MS data to export (abf) format were packaged in MS-DIAL 4.61 an enhanced standardized untargeted lipidomics and metabolomics by using (MSP) format libraries databases^17^.

### Pharmacological study

#### In vitro study

##### Antioxidant activity (DPPH and ABTS methods) of different MA wastes

DPPH and ABTS free radical-scavenging activity method was adopted to measure the in vitro antioxidant activity of MA ethanolic, butanolic, ethylacetate, chloroform and petroleum ether extract; at different concentration (15.62, 31.25, 62.5, 125, 250 and 500 μg/mL) of the three parts extract; ascorbic acid and trolox was used as a positive control. The radical scavenging model for antioxidant activity, using 1,1-diphenyl-2-picrylhydrazyl (DPPH, 250 mM), was performed according to Shimada et al.^18^. ABTS^+^ dissolved in water to a 7 mM concentration. ABTS stock solution with 2.45 mM potassium persulfate (final concentration) and allowing the mixture to stand in the dark at room temperature for 24 h before use Oxidation of the ABTS was performed according to Dinkova-Kostova et al.^19^. The inhibition of the DPPH and ABTS radical were calculated using the following formula: % Inhibition = [(A control − A sample)/A control] × 100. Where; A is the absorbance at 517 nm in DPPH and 734 nm in ABTS by using UV spectrophotometer (Agilent Technologies Carry 100 UV–Vis) was used for absorption measurements.

#### In vivo study for anti-ulcerative colitis activity

##### Materials

*Animals* The present study used male Wistar albino rats, of body weights (bwt) (150–175 g) obtained from the animal house colony of the National research centre, Dokki, Giza, Egypt. The rats were kept in standard metal cages in an air conditioned room at 22 ± 3˚C, 55 ± 5% humidity and provided with standard laboratory diet and water ad libitum. The present study complies with local and national guidelines, as it was done in accordance with the guide for care and use of laboratory animals and obtained ethics committee approval certificate from National Research Centre ethics committee numbered 16/138. Experiments were performed according to the National Regulations of Animal Welfare and the Institutional Animal Ethical Committee (IAEC) in Egypt,and is reported in accordance with Animal Research: Reporting of In Vivo Experiments (ARRIVE) guidelines.

*Drugs and chemicals* (a) Prednisolone (Sigma Chemical Co.,St Louis, MO, U.S.A.) used as reference anti-inflammatory drug and was given orally by gastric tube. (b) Acetic acid (Elgomhoreya Co, Cairo, Egypt) injected per rectum (Pr) for induction of colonic inflammation and ulceration; a model mimicking ulcerative colitis. (c) Diethyl ether (Sigma Chemical Co., St. Louis, MO, USA), for animal anaesthesia during blood sampling from retro-orbital plexus of veins and for euthanasia under anaesthesia. (d) Formaldehyde (Sigma Chemical Co., St. Louis, MO, USA), for fixation of postmortem tissues dissected for histopathologic examination.

*Diagnostic kits* (a) Kits for determination of Liver function tests (aspartate and alanine aminotransferase) and Kidney function tests (urea and creatinine) in serum were purchased from Biodiagnostic company, Dokki, Giza, Egypt. (b) Serum highly sensitive C-Reactive Protein (CRP) was measured according to the manufacturer kit using rat hs-CRP ELISA kit from Wuhan Fine Biotech. Ltd. Co., China. (c) Serum Interleukin-6 was measured according to the manufacturer kit using rat IL-6 ELISA kit Cat. no. ER0042 from Wuhan Fine Biotech. Ltd. Co., China. (d) Serum Anti-Neutrophil Cytoplasmic Antibodies (ANCA)was measured according to the manufacturer kit using rat ANCA ELISA kit Cat. no. SL1417Ra from SUNLONG Biotech. Ltd. Co., China.

##### Methods

In vivo biological studies were conducted to investigate some pharmacological activities of anti-inflammatory and colonic anti-ulcerative activities of MA fruit peels, leaves and pseudo-stem extracts, after ensuring their safety.

*Acute and subchronic toxicity studies* Determination of safety of the tested herbal extracts was done by performing acute and subchronic toxicity studies.

*Experimental design acute toxicity* The butanol extracts of MA leaves, pseudo-stem and fruit peels were dissolved in distilled water then given orally (Po) in a dose of 5000 mg/kg to three groups of rats each consisted of five rats. A fourth group acted as negative control group and received the same volume of distilled water. The percentage of mortality was recorded 24 h later. No mortality occurred after 24 h. Close monitoring of animals’ change in body weight, bowel habits, hair colour or behaviour, was noticed during the next 2 weeks^20^.

*Subchronic toxicity* According to the results of acute toxicity study the selected doses for chronic toxicity study were 250 and 500 mg/kg.

Fifty six male rats were classified equally into seven groups: Negative control group given one ml of distilled water orally (Po). Treated groups received different three parts MA leaves, pseudo-stem, fruit peels extract in two doses 250,500 mg/kg, All extracts were given orally for 14 days.

*Detection of the effect of treatment on body weight* All rats in all groups were weighed before starting the experiment, after acute toxicity study and after subchronic toxicity study. Precaution was taken that the amount of daily chaw was fixed and equal for all groups.

*Biochemical parameters* Two days after ulcer induction, The animals in all groups were kept fasting for 12 h, on the fifteenth day of the subchronic study, blood was obtained from all groups of rats after being lightly anaesthetized with ether by puncturing retro-orbital plexus, the blood was allowed to flow into a clean dry centrifuge tube and left to stand 30 min before centrifugation for 15 min at 2500 rpm with RCF = 1048 gf, to avoid haemolysis^21^. The clear supernatant serum was separated and collected for determination of serum levels of liver function tests (aspartate and alanine aminotransferase) according to Reitman and Frankel^22^; blood urea and serum creatinine were measured by the methods described by Patton and Crouch^23^ and Young^24^, respectively.

*Efficacy study: Experimental design* Seventy two male Wister albino rats were divided equally into 9 groups as follows: Negative control group given one ml of distilled water orally (Po). Positive control group for which colonic inflammation and ulceration were induced without previous treatment. MA pretreated groups received leaves extract (250, 500 mg/kg), pseudo-stem extract (250, 500 mg/kg) and fruit peels extract (250, 500 mg/kg). The six MA pretreated groups were given the extracts orally for two successive weeks, which is the same duration of pretreatment with the standard drug prednisolone. Prednisolone pretreated group in a dose of 5 mg /kg given orally for two successive weeks^25,26^. The last dose of each treatment was administered 2 h before ulcer induction. The selected efficacy experimental dose used in the present study depended on the results of the acute toxicity and subchronic toxicity studies which had proven the safety of tested extracts.

*Method of induction of colonic ulcer* All rats were fasted overnight with access to water only, before being anesthetized with ether inhalation. A polyethylene catheter (2 mm diameter) was inserted 8 cm into the lumen of the colon via the rectum (Pr). For all treated and positive control groups, an acetic acid solution (2 mL, 8%, v/v in saline) was slowly infused into the colon through the catheter. The acid solution was then aspirated and 2 mL of phosphate buffer solution (pH = 7) was infused into the rectum of each rat^27^. Negative control rats received an equi-volume saline solution devoid of acetic acid.

*Biochemical parameters* Two days after ulcer induction, blood sampling and centrifugation was done^21^. The clear supernatant serum was separated and collected for determination of serum levels of liver function tests (aspartate and alanine aminotransferase) according to Reitman and Frankel^22^; blood urea and serum creatinine were measured by the methods described by Patton and Crouch^23^ and Young^24^, respectively. systemic inflammatory marker C-reactive protein (CRP) and determination of interleukin beta six (ILβ6) and ANCA following manufacturer’s instructions according to the methods described by Sibiya et al.^28^; Sen et al.^29^ and Hauschild et al.^30^ respectively.

*Macroscopic examination* All animals were sacrificed with ether and laparotomy was performed. Colonic segments (8 cm in length and 3 cm proximal to the anus) were excised, opened along the mesenteric border, washed with saline, and scored macroscopically. Gross mucosal lesions were recognized as hemorrhage or erosions with damage to the mucosal surface. The number and severity of mucosal lesions were noted and lesions were scaled as follows: Almost normal mucosa = 0, Petechial lesions = 1, one or two lesions or lesions less than 1 mm = 2, severe lesions or lesions between 1 and 2 mm = 3, very severe lesions or lesions between 2 and 4 mm = 4, Mucosa full of lesions or lesions more than 4 mm = 5. Mean ulcer score for each animal was expressed as ulcer index (U.I) and the percentage of inhibition against ulceration was determined using the expressions in the following equation^31,32^:

$${\text{UI}}\, = \,{\text{UN}}\, + \,{\text{US}}\, + \,{\text{UP}}\, \times \,{1}0,$$

UI = Ulcer index, UN = Ulcer number, US = Ulcer severity, UP = Percentage of ulcerated colons.

$$\% {\text{Ulcer}}\,{\text{inhibition}}\, = \,{1}00 - \left[ {{\text{U}}.{\text{I}}.\,\;{\text{in}}\;\,{\text{pretreated rats}}/{\text{U}}.{\text{I}}.\,\;{\text{in}}\;{\text{positive control}}} \right]\, \times \,{1}00.$$

*Histological assessment of liver and kidney tissue for MA extracts acute and sub-chronic toxicity studies and Colon mucosa for efficacy of the MA extracts in anti-ulcerative colitis study* Different sections from the liver, kidneys and colon were cut and fixed in 10% formalin. The tissues were then dehydrated in ethanol and embedded in paraffin bocks. The liver and kidney tissues were cut into sections of 4-μm thickness, while the colon tissues were cut into 5 µm thick sections. All tissues were stained with hematoxylin and eosin (H&E), and conventional histopathological examination was carried out under light microscopy by a pathologist who was blinded to the therapeutic strategy. Images were acquired with a Leica ICC50 HD digital camera attached to a Leica motorized light microscope system^33^.

For assessment of colonic tissue damage, the tissue sections were examined blindly and the lesions were semiquantitively evaluated in ten random low power fields, as described by Amir Rashidian et al.^34^. The grading system is scaled from 0 to 5 and the details of this grading system are illustrated in Table 1^34^.

**Table 1:** Pathological grading system of colonic ulcers.

Grades	Histopathological criteria
Grade 0	Normal histological structure of colonic mucosa, submucosa and T.muscularis with no inflammatory cell infiltration
Grade1	Infiltration of the colonic mucosa and submucosa with Inflammatory cells
Grade 2	Infiltration of the colonic mucosa, submucosa and T.muscularis (trans-mural) with Inflammatory cells
Grade 3	Focal ulceration with trans-mural inflammation
Grade 4	Multiple large ulcers with trans-mural inflammation
Grade 5	Extensive ulcerations with complete necrosis of the colonic mucosa and intense trans-mural inflammation

*Immunohistochemistry* Immunohistochemical procedures for the demonstration of myeloperoxidase immune reactivity, a marker of neutrophil infiltrstion, in the colon were performed according to the method of Hassan et al.^33^. Briefly, the paraffin-embedded colon sections were deparafinized and rehydrated in ethanol. The sections were then incubated with rabbit monoclonal anti-myeloperoxidase antibody (ERP20257, Abcam). The sections were stained with diaminobenzidine (DAB) for the demonstration of the immune reaction. Finally, counterstaining with hematoxylin was carried out. MPO immunohistochemical staining was semi quantitively assessed in the colonic mucosa and submucosa in ten random high microscopic power fields, according to the % of positively stained cells. A grading system scaled from 0 to 4 was used; in which 0 = no immune staining; 1 = positive staining in ˂ 25% of cells in HPF; 2 = positive staining in 25–50% of cells in HPF; 3 = positive staining in 51–70% of cells in HPF; and 4 = positive staining in ˃ 70% of cells in HPF^33^. The assessment of immunohistochemical analysis was performed using image J 1.8.0 (https://imagej.nih.gov/ij/download.html).

*Statistical analysis* The data were expressed as means ± SE for each group. Results were analyzed using one-way analysis of variance, followed by the Tukey–Kramer test for multiple comparisons; P value of less than 0.05 was considered significant in all types of statistical tests. Graph Pad Software (Graph Pad Software Inc., La Jolla, CA, USA) (version 6) was used to carry out the statistical tests.

## Results and discussions

### Phytochemical study

Musa Wastes are a potential source of bioactive metabolites that’s why they were subjected to phytochemical and pharmacological studies for evaluating their anti-ulcerative colitis effects. They provided a considerable promising supplement against ulcerative colitis (Fig. S1).

#### Isolation and identification of metabolites

The crude leaf extract of MA (70% hydroalcoholic) was applied to polyamide column chromatography and eluted with water methanol mixtures in the order of decreasing polarity.

All collected fraction was investigated individually by TLC chromatography.

**Figure 1:**
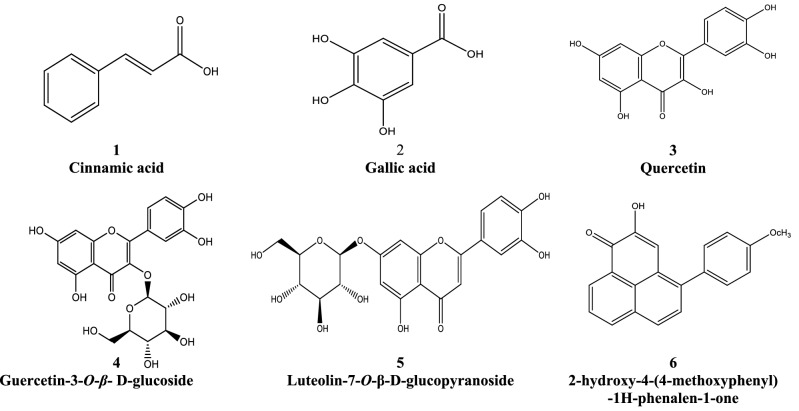
Six isolated compounds two phenolic acids, one phenylphenalenone and three flavonoids.

The 20–50% methanolic/water subfractions uploaded on polyamide 6 column using water/methanol in order of decreasing polarity. The collected subfractions was purified by Sephadex LH-20 using 50% ethanol/water as eluent to yield 5 compounds identified as, quercetin, quercetin-3-O-*β*-d-glucoside, luteolin-7-*O*-*β*-d-glucopyranoside and Gallic acid, cinnamic acid. On the other hand, the 70% alcohol subfraction was applied to Sephadex LH-20 column chromatography and eluted with 80% ethanol/water to give one pure compound as 2-hydroxy-4-(4-methoxyphenyl)-1H-phenalen-1-one (Fig. 1).

These compounds were identified by comparing their spectral data with those reported.

**Compound 1** *was identified as trans-cinnamic acid* It is soluble in chloroform and methanol. Moreover, **Compound 1** gives blue color under UV light at 254 nm with R_F_: 0.87, ^1^H NMR (CD3OD, 400 MHz): δ ppm = 7.63(d, J = 15.9, 1H, H-β), the remaining phenyl ring resonate at δppm 7.61–7.57 (m, 2H) and 7.42–7.38 (m, 3H), 6.51 (d, J = 15.9, 1H, H-α) and ^13^C NMR (CD_3_OD, 400 MHz): δ ppm :171.65 (C=O), 145.05 (C-β), 136.16 (C-1), 131.05 (C-4), 129.93 (C2 & C-6), 129.00 (C-3 & C-5), 120.99 (C-α) in (Fig. S2 and Table 2)^35^.

**Table 2:** HPLC and LC-MSMS data in low and high resolution of different MA parts identified by tentative mass.

No	Tentative identification	RT	Chemical formula	Low mass	High resolution mass			UV λ_max_	*Musa acuminata*		
				[M−H]^−^	Measured and caculated	mass of [M−H]^−^	∆ ppm		1	2	3
**Phenolic acids and phenolic glycosides**											
1	Vanillin	1.63	C_8_H_8_O_3_		151.0397, 151.0390	136.0152, 108.0202, 93.0332, 69.0702	− 1.7152	216, 259	*_878.06_	*_23.17_	–
2	l-Tyrosine methyl ester	2.04	C_10_H_13_NO_3_	194.25, 176.24, 128.23, 84.43	194.0812, 194.0812	176.0706, 162.0561, 118.1188, 96.0562	0.0361	206, 263	*	*	–
3	Bergapten^a^	2.12	C_12_H_8_O_4_		215.0320, 215.0339	179.0555, 131.0447, 113.0228, 89.0227, 71.0121	7.4296	206,224	*	–	*
4	Quinic acid^a^	2.15	C_7_H_12_O_6_		191.0551, 191.0550	173.0444, 127.0386, 111.0072, 85.0279, 59.0122	0.6044		*	–	*
5	Threonic acid^d^	2.14	C_4_H_8_O_5_		135.0285, 135.0293	117.0179, 89.0227, 75.0071	− 6.3694	202, 220	*	–	
6	Caffeoyl quinic acid^a^	2.17	C_16_H_18_O_9_		353.0853, 353.0867	263.0588, 173.0443, 155.0337, 113.0228, 89.0227	− 3.9987	202	*	–	*
7	N-Fructosyl isoleucine^d^	2.18	C_12_H_23_NO_7_		292.1401, 292.1403	227.0654, 203.0667, 173.0555, 141.0655, 130.0859	3.497	220	*	–	*
8	N-Fructosyl pyroglutamate^d^	2.40	C_11_H_17_NO_8_		290.0883, 290.0884	254.0660, 212.0555, 200.0555, 170.0447, 128.0338	− 0.2154	216	*	–	*
9	Tyrosine^d^	2.52	C_9_H_11_NO_3_		180.0656, 180.0655	163.0389, 119.087, 93.0330	0.6865	262, 357	*	–	*
10	Methoxytyrosine^d^	3.64	C_10_H_13_NO_4_		210.0765, 210.0761	128.0389, 118.0647, 94.0282, 66.0332	2.1598	221.335	*	*	*
11	Acetylleucine^d^	3.86	C_8_H_15_NO_3_		172.0965, 172.0968	130.0859, 59.0803	− 1.6122	209, 259, 335	*	*	*
12	Tryptophan^d^	3.88	C_11_H_12_O_2_N_2_		203.0818, 203.0815	142.0652, 116.0489, 97.0283	1.2432	215, 239, 258	*	*	–
13	Gallic acid^b,C^	2.66	C_7_H_6_O_5_		169.0132, 196.0131	125.0229, 97.0281, 65.0016	0.2002	258. 288, 373	* ^10.01^	–	* ^20.01^
14	Salicylic acid^d^	2.93	C_7_H_6_O_3_		137.0232, 137.0239	93.0330	− 4.7078	220	*	*	*
15	Benzoic acid^d^	3.56	C_13_H_16_O_9_		315.0717, 315.0711	165.0176, 152.0101, 132.0005, 108.0200, 85.0276	2.0880	202, 252	*	*	–
16	Cinnamyl alcohols ^a^	3.78	C_9_H_10_O_3_		165.0546, 165.0546	147.0439, 119.0487, 72.9915	− 0.0573	262, 319, 380	*	–	*
17	Caffeoylquinic acid^a^	3.91	C_16_H_18_O_9_		353.0876, 353.0880	191.0562, 180.0377, 161.0228, 135.0435	2.525	254, 348	*	*	*
18	Caffeic acid^b,a^	4.00	C_9_H_8_O_4_		179.0342, 179.0339	135.0437, 103.9188, 71.0122	1.9857	241, 335	–	*_25.46_	*_106.27_
19	P-Coumaric acid^b^	4.09	C_9_H_8_O_3_		163.0389, 163.0395	119.0488	− 4.0167	245	–	* ^162.91^	* ^17.16^
20	Ferulic acid^b,C^	4.11	C_10_H_10_O_4_		193.0492, 193.0495	178.0259, 149.0599, 134.0357	1.6080	241, 331	* ^29.76^	–	* ^59.60^
21	Citric acid^a^	4.22	C_6_H_8_O_7_	190.20, 110.26	191.0184, 191.0186	111.0071, 87.0070	− 1.4275	241, 320	*	–	–
22	Propyl gallate^b^	4.25	C_10_H_12_O_5_		211.0242, 211.0237	167.0339, 123.0437, 93.0330, 65.0381	2.1586	–	* _F4713.85_	* 34.70	* 20.50
23	Vanillic acid hexoside^a^	4.91	C_14_H_18_O_9_		329.0884, 329.0867	195.1921, 167.0340, 109.0281	5.0758	202, 209	*	*	*
24	Sinapic acid-*O*-glucoside^a^	4.97	C_17_H_22_O_10_		385.1148, 385.1166	263.0767, 223.0605, 205.1228, 173.0441, 153.0909	4.6739	241, 335	*		*
25	Cinnamic acid^b^	5.39	C_9_H_8_O_2_		147.0444, 147.0441	–	2.2565	256, 356	*_45.91_	*_61.32_	*_83.63_
26	4-*O*-Feruloylquinic acid^a^	5.58	C_17_H_20_O_9_		367.1024, 367.1024	193.0498, 173.0443, 134.0358, 93.0327	0.0014	263, 349	*	*	
27	Kaempferol-3-*O*-Sophortrioside^a^	5.86	C_33_H_40_O_21_	771.17, 609.08, 300.44, 178.20	771.2281, 771.2289	609.1613, 301.0349	1.0373	241, 263	*		*
28	Quercetin 3-*O*-glucosyl-glucoside^a^	5.90	C_30_H_26_O_15_	625.12, 462.90, 300.50	625.1530, 625.1550	463.0885, 301.0351	3.1992	241,335	*		
29	Diosmetin-7-*O*-rutinoside^a^	6.31	C_28_H_32_O_15_	607, 299.46, 270.41	607.1668, 607.1685	299.0199, 270.0160	2.7998	241, 267, 310	*	*	*
30	Kaempferol-3-*O*-rutinoside^a^	6.35	C_27_H_30_O_15_	593.09, 284.45, 254.35, 189.16	593.1531, 593.1501	485.1238, 388.0862, 285.0401	5.0235	241, 270, 310	*		
31	Peonidin 3-rutinoside^a^	6.39	C_28_H_33_O_15_	608.12, 341 63	608.1401, 608.1470	343.0461, 300.0273, 271.0236, 178.9975, 125.0230	11.3460	241, 270, 310	*	*	
32	Sulfo jasmonate^d^	6.41	C_12_H_18_O_7_S		305.0699, 305.0690	255.1124, 96.9585	2.9656	241, 306, 349		*	*
33	Luteolin-7-*O*-*β*-d-glucopyranoside^b,d^	6.48	C_21_H_20_O_11_		447.0929, 447.0922	285.0405	1.5147	241, 320	* _210.21_	* 50.23	* 120.56
34	Delphinidin 3-rutinoside^a^	6.53	C_27_H_31_O_16_		610.1607, 610.1603	301.0324, 272.0288, 151.0024	− 0.6555	241,270, 306, 320	*	–	–
35	Quercetin *O*-rhamnoside-*O*-hexoside^a,b^	6.56	C_27_H_30_O_16_	609. 11, 300.48, 242.59	609.1554, 609.1560	343.0462, 301.0716, 242.0568, 151.0023	0.9849	241, 270	*	–	*
36	Naringin^b^	6.55	C_27_H_32_O_14_	579.13, 458.95, 270.45	579.1782, 579.1771	459.1146, 402.1311, 339.0713, 271.0612, 235.0239, 181.0490, 151.0023	− 1.8992	241, 320	*_470.93_	*_19.16_	–
37	Quercetin-3-O-glucoside^C^	6.65	C_21_H_20_O_12_	462.96, 300.46	463.0886, 463.0871	300.0273, 271.0241, 151.2179	3.2179	241, 277, 320	*	*	*
38	Quercetin-3-O-rutinoside^a,b^	6.70	C_27_H_30_O_16_	609.08, 300.44	609.1576, 609.1580	325.700, 301.0707, 242.0578, 151.0020	0.6566	241, 277, 320	*_3806.15_	*_480.14_	*_50.08_
39	isorhamnetin-3-O-galactoside-6″-rhamnoside^a^	6.76	C_28_H_32_O_16_	623.12, 314.56, 466.90	623.1368, 623.1370	463.0908, 357.0613, 314.0431, 271.0251, 151.0024	0.3209	241, 270, 320	*	*	–
40	Kaempferol-3-O-glucoside^a^	6.79	C_21_H_20_O_11_	446.97, 283.42, 254.42, 150.15	447.0931, 447.0922	284.0325, 255.0300, 151.0031	2.0608	241, 320	*	*	*
41	Apigenin-7-O-neohesperidoside^a^	6.94	C_27_H_30_O_14_	577.87	577.1615, 577.1617	460.0598, 269.0454, 175.0395	0.3465	241, 270, 306	*	-	*
42	isorhamnetin-3-O-glucoside^a^	7.08	C_22_H_22_O_12_	476.96, 356.58, 313.61, 242.32	477.1044, 477.1028	357.0601, 314.0432, 243.0270, 258.0270, 151.0027	3.4546	241, 270, 320	*	-	*
43	Dodecyl sulfate^a^	7.33	C_12_H_26_O_4_S		265.1479, 265.1468	219.8447, 185.1164,96.9584	3.9646	241, 270, 306	*	–	–
45	2-Methoxycinnamic acid^d^	7.67	C_10_H_10_O_3_		177.0545, 177.0546	162.0311, 145.0279, 121.0277	− 0.7429	241, 306	*	–	*
46	Daidzein^b^	7.64	C_15_H_10_O_4_		253.1444, 253.1434	191.14440, 125.0956	3.6650	241, 267	–	*_14.22_	–
47	Aloe-emodin^d^	7.76	C_15_H_10_O_5_		269.0456, 269.0444	225.1853	4.1364	246, 275	*	–	–
48	Catechin^b,C^	8.12	C_15_H_14_O_6_		289.0507, 289.0495	245.0601, 217.0650	3.9939	243, 281	*^908.23^	–	–
49	Delphinidin^d^ 7-hydroxyflavonoids	8.20	C_15_H_11_O_7_		301.0354, 301.0343	273.0391, 178.9977, 151.0023, 121.0278	2.8125	241, 277, 320	–	*	*
50	Quercetin^b,C^	8.35	C_15_H_10_O_7_		301.0356, 301.0343	178.9977, 151.0025, 121.0277, 83.0123	3.8263	245	* ^1222.64^	* ^379.66^	* ^183.59^
51	Atractylenolide III^d^ (sesquiterpenoid)	8.80	C_15_H_20_O_3_		247.1336, 247.1329	203.1433, 185.1327, 169.1007	3.0975	241, 306	*	–	*
52	Wogonin^d^ (8-O-methylated flavonoids)	11.15	C_16_H_12_O_5_		283.0611, 283.0601	268.0374, 157.0088	3.5196	–	*	–	*
53	Kaempferol-4′-methyl ether^d^ (flavonols)	9.00	C_16_H_12_O_6_		299.0562, 299.0550	284.0326, 256.0372	4.1108	241, 310, 403	*	–	*
54	Kaempferol^a^	9.13	C_15_H_10_O_6_		285.0405, 285.0394	151.0033	4.0797	245, 310	–	*	*
**Phenylphenalenones**											
56	2-(4^/^-hydroxyphenyl)-1,8-naphthalic anhydride	9.20	C_18_H_10_O_4_		289.0507, 289.0495	245.0610, 221.1175, 176.1829	3.9939	245, 270	*		
57	Musanolone E	9.23	C_19_H_12_O_4_		303.0662, 303.0652	285.0555, 259.0756	3.3237	245, 270	*		
58	2,3-Dihydro-4-(4-methoxyphenyl)-1H-phenalene-1,2,3-triol	9.50	C_20_H_18_O_4_		321.1129, 321.1121	306.0895, 212.0462, 199.1693	2.3325	245, 277	*		
59	2,3-dihydro-2,3-dihydroxt-4-(4^/^-hydroxyphenyl) phenalen-l-one	9.76	C_19_H_14_O_3_		305.0813, 305.0808	277.0876, 249.0900, 180.0665, 108.0201	1.6191	245, 277	*	–	*
60	2-hydroxy-4-(4-methoxyphenyl)-1H-phenalen-1-one	9.93	C_20_H_14_O_3_		301.0861, 301.0859	286.0634, 258.0685, 176.1632	0.6636	245, 277	*	–	–
61	Irenolone	9.95	C_19_H_12_O_3_		287.0713, 287.0703	259.0766, 107.0167	3.6536	245, 270	*	–	–
62	Anigorufone	10.13	C_19_H_12_O_3_		287.0713, 287.0703	259.0762, 138.1300	3.4410	–	*		*
63	Hydroxyanigorufone	10.96	C_19_H_12_O_2_		271.1807, 271.1798	253.1807, 209.1911	3.5101	–	*		*
**Fatty acid, triterpenes and lipids**											
64	Isopropylmalic acid^d^ |Hydroxy fatty acids^d^	3.77	C_7_H_12_O_5_		175.0600, 175.0601	131.0697, 115.0385, 85.0642	− 0.4976	241, 306	*	-	
65	Azelaic acid^d^	5.44	C_9_H_16_O_4_		187.0966, 187.0965	125.0958, 111.0072, 97.0643	0.5488	242, 326	*		
66	Hydroxysebacic acid^d^	5.97	C_10_H_18_O_5_		217.1074, 217.1071	171.1016, 155.1063, 88.1209	1.5718	241, 335	*		
67	FA 18:2 + 4O^d^ Long-chain fatty acids	7.48	C_18_H_32_O_6_		343.2124, 343.2115	292.9891, 211.1328, 59.0123	2.6739	241, 277, 306	*		
68	A 18:2 + 3O^d^ |Lineolic acids and derivatives	8.49	C_18_H_32_O_5_		327.2176, 327.2166	309.2063, 291.1964, 229.1443, 211.1334, 181.1380, 171.1015, 137.0952, 113.0243, 85.0279	3.0249	241, 277, 306	*	*	*
69	FA 18:1 + 3O^d^ |Long-chain fatty acids	8.52	C_18_H_34_O_5_		329.2332, 329.2323	314.0430, 259.1550, 229.1433, 171.1011, 101.0676	2.8376	241, 277, 306	*		
70	FA 18:1 + 2O^d^	8.88	C_18_H_34_O_4_		313.2385, 313.2373	285.0403, 178.9978, 155.1066	3.6997	249, 275	*	*	*
71	FA 18:4 + 2O^d^ |Lineolic acids and derivatives	8.95	C_18_H_30_O_4_		309.2053, 309.2060	291.1965, 221.1532, 209.1177, 195.1019, 171.1018, 113.0950	-2.5053	249, 272	*	*	*
72	FA 18:4 + 2O^d^ |Lineolic acids and derivatives	9.46	C_18_H_28_O_4_		307.19186, 307.18979	289.1808, 259.1724, 235.1337, 211.1334, 185.1170, 121.0642	2.07	245, 349	*		*
73	FA 18:1 + 1O^d^ Lineolic acids	9.97	C_18_H_34_O_3_		297.2434, 297.2424	297.2329, 253.2476, 183.0114, 155.1065, 127.1114	3.2172	272	*		*
74	FA 18:3 + 1O^d^	11.11	C_18_H_30_O_3_	292.69, 274.55, 223.39, 194.36	293.2123, 293.2111	275.2015, 223.1699, 211.1334, 183.1018, 171.1016, 155.1068, 121.1008	3.9528	249	*	*	*
75	LPE 16:0^d^	11.73	C_21_H_44_NO_7_P		452.2786, 452.2785	294.9081, 255.2330, 234.1745, 92.9932		–	*	*	*
76	Corosolic acid^d^ (triterpenoids)	12.39	C_30_H_48_O_4_		471.3472, 471.3469	354.3522	0.6621	–	–	*	*

Compounds listed in the Table were found in (1) total extract of *leaves MA*, (2) Pesudostem, and (3) Fruit Peels, (a) compounds compared with literature ^7,40–43^, (b) compounds identified from Standard Authentic compound Quantitative with HPLC (conc. as µg/g), (c) compounds identified from NMR , (d) compounds identified from DataBase (MS-Dial, *KNApSAcK*, Metlin, and *RIKEN*).

**Compound 2** *was identified as gallic acid* It soluble in methanol and gave light blue color under UV at 254 nm. ^1^H NMR analysis (400 MHz, DMSO-*d*_*6*_) *δ* ppm: 6.97 (S). ^13^C NMR: (*δ* ppm) 165.67 (C7; C=O) 144.10 (C3 & C5), 138.99 (C4), 118.82 (C1) 108.86 (C2 & C6) (Fig. S3 and Table 2)^36^.

**Compound 3** was identified as quercetin; It is soluble in methanol and give yellow color under UV light, 356 nm, ^1^H NMR (400 MHz, DMSO): δ 7.74 (1H, d, J = 2.1 Hz, H-2′), 7.62 (1H, dd, J = 8.3, 2.1 Hz, H-6′), 6.88 (1H, d, J = 8.3 Hz, H-5′), 6.39 (1H, d, J = 2.0 Hz, H-8), 6.18 (1H, d, J = 2.0 Hz, H-6)” and ^13^C NMR: δ (ppm) 93.2 (C-8), 98.0 (C-6), 102.9 (C-10), 115.0 (C-2′), 115.4 (C-5′), 119.8 (C-6′), 121.8 (C-1′), 135.5 (C-3), 144.9 (C3′), 146.7 (C-2), 147.5 (C-4′), 156.0 (C-9), 160.6 (C-5), 163.8 (C-7), 176.7 (C-4). Compounds 1,2 and 3 were eluted with mobile phase benzene:methanol:acetic acid, 45:8:4 (Fig. S4 and Table 2)^37^.

**Compound 4** *was identified as quercetin-3-O-β-*d*-glucoside*; it soluble in methanol gave dark purple color under UV light at 254 nm with R_F_: 0.42 eluted with mobile phase butanol:acetic acid:water, 30:10:10. ^1^H NMR (400 MHz, DMSO-d6: 7.59 (1H, d, J = 1.8, H-2), 7.62 (1H, dd, J = 1.8 & 8.4, H-6′), 6.87 (1H, dd, J = 8.4, H-5′), 6.39 (1H, d, J = 2.2, H-8), 6.19 (1H, d, J = 2.4, Í-6), 5.43 (1H, d, J = 7.6,H-1″13C NMR spectrum (100 MHz, DMSO-d6, ppm): 156.39 (C-2), 133.31 (C-3), 178.18 (C-4), 161.12 (C-5), 98.66 (C-6), 163.23 (C-7), 94.13 (C-8), 156.14 (C-9), 104.75 (C-10), 120.88 (C-1′), 117.04 (C-2′), 145.25 (C-3′), 148.78 (C-4′), 114.28 (C-5′), 122.01 (C-6′), 101.09 (C-1″), 74.16 (C-2″), 76.73 (C-3″), 70.89 (C-4″), 77.65 (C-5″), 61.18 (C-6″) (Fig. S5 and Table 2)^37^.

**Compound 5** *was identified as luteolin-7-O-β-*d*-glucopyranoside* It soluble in methanol and give dark purple under UV lamb at 254 nm with R_F_: 0.891 eluted by mobile phase butanol:acetic acid:water, 30:10:10. ^1^H-NMR (DMSO-*d*_*6*_) revealed signals at *δ* ppm 7.45 (*dd*, *J* = 8.3 Hz, and *J* = 2.2 Hz, H-6′), 7.42 (*d*, *J* = 2.2 Hz, H-2′), 6.9 (*d*, *J* = 8.3 Hz, H-5′), 6.8 (*d*, *J* = 2.2 Hz, H-8), 6.7 (s, H-3), 6.4 (*d*, *J* = 2.2 Hz, H-6) and signal appeared as doublet at *δ* ppm 5.08 (*d*, *J* = 6.6 Hz, H-1″ of glucose) assignable for the anomeric proton of the sugar moiety and ^13^C-NMR spectrum (DMSO-*d*_*6*_). ^13^C NMR (100MHZ, DMSO-d_6_) δ (ppm): 181.92 (C-4), 164.99 (C-7), 163.14 (C-2), 161.51 (C-5), 157.97 (C-9), 149.95 (C-4′), 147.12 (C-3′), 118.88 (C-6′), 122.12 (C-1′), 116.59 (C-5′), 113.86 (C-2′), 103.89 (C-10), 103.19 (C-3), 99.82 (C-1′′), 99.58 (C-6), 94.49 (C-8), 77.14 (C-5′′), 76.71 (C-3′′), 74.02 (C-2′′), 70.11 (C-4′′), 61.17 (C-6′′) (Fig. S6 and Table 2)^38^.

**Compound 6** *was identified as 2-hydroxy-4-(4-methoxyphenyl)-1H-phenalen-1-one*, it soluble in chloroform and gave light blue under UV lamb, 254 nm. with R_F_: 0.82 eluted by mobile phase methanol: chloroform, 9.5:0.5.^1^H NMR (400 MHz, CDCl_3_), δppm: 392(3H s, OCH_3_,)7.05 (2H, d, J = 8.48 Hz, H-3′ and H-5′), 7.34(1H, s, H-3), 7.42 (2H, d, J = 8.48, H-2′ and H-6′),7.58 (1H, d, J = 8.49, H-5), 7,81(1H, t, J = 8.49 Hz, H-8), 7.95 (1H, d, J = 8.49, H-6), 8.27 (1H, dd, J = 1.2 & 8.3 Hz, H-7), 8.78 (1H, dd, J = 1.2 &8.3, H-9); ^13^CNMR(CDCI_3_ ppm: 55.60 (OCH_3_), 112.89 (C-3), 114.15 (C-3′ and C-5′), 125.04 (C-9b), 126.63(C-3a), 127.77(C-8), 129.84 (C-9a), 130.14 (C-5), 131.38 (C-6), 131.65(C-6a), 131.72(C-9). 132.00 (C-2′ and C-6′), 132.39 (C-1′), 136.64 (C-7), 143.99 (C-4), 149.49 (C-2), 159.75(C-4′), 179.98 (C-l) (Fig. S4 and Table 2)^39^.

#### GC–MS of essential oil from fruit peels

The essential oil was extracted from fruit peels by hydrodistillation for five fours which is the suitable time to obtain the volatile oils.

**Figure 2:**
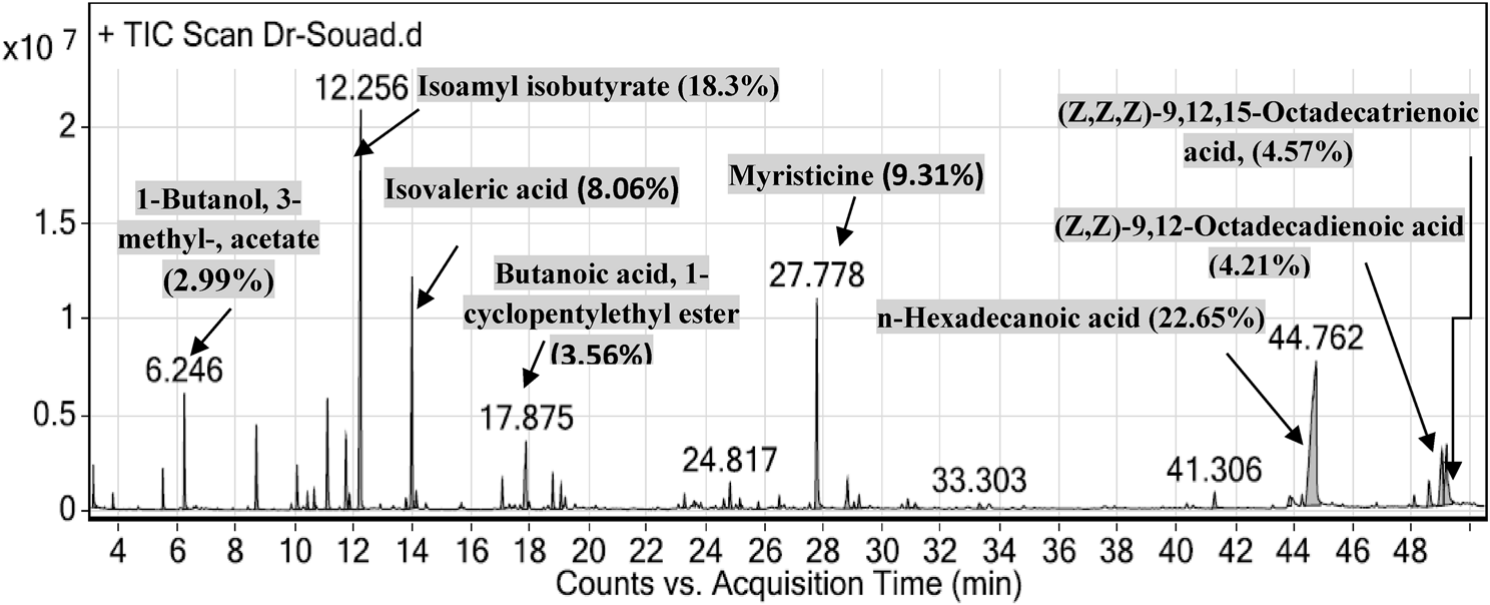
GC/MS chromatogram of the *Musa acuminata* essential oil fruit peels and their main constituents.

Injection of the essential oil to GC/MS led to identification of 37 compounds. The major compounds were isoamyl isobutyrate (18.3%) and n-hexadecanoic acid (22.05%) followed by myristicine 9.31% and isovaleric acid amounted to 8.06% of the oil. Occasionally, our results are relatively similar to that identified from banana reported by Facundo et al.^40^. These constituents of fruit peels were presented in (Fig. 2 and Table S3).

### HPLC and LC-MSMS profiles of secondary metabolites from MA

The metabolomics profile was identified based on low and high throughput sensitive LC/MS analyses which enabled the in-depth studies of secondary metabolite changes in MA plant with different parts as leaves, pesudostem and in fruit peels. 76 different compounds were identified including phenols, flavonoids, phenylphenalenones, amino acids and fatty acids from the agro waste of different parts of MA. LC-MSMS profile was used as a marker for the ulcerative colitis. The individual compounds were identified via comparison of the exact molecular masses (**∆** less than 5 ppm, mass spectra and retention times) with those of the standard compounds available in PubChem, ChEBI, Metlin, KNApSAck, HPLC, NMR and literature data. Different types of phenolic compounds of MA extracts were recorded in (Table 2) included phenolic acids and polyphenols such as gallic acid, caffeic acid, syringic acid, ferulic acid, Salicylic acid, Caffeic acid, Caffeoylquinic acid, kaempferol, catechin, Feruloylquinic acid, Vanillic acid hexoside, Sinapic acid-*O*-glucoside, and Kaempferol 3-Sophortrioside.

The biologically effects of MA extract are most probably due to its content found in the different extracts. These fractions included the petroleum ether, chloroform fraction, ethyl acetate fraction, n-butanol fraction, and water fraction. In the biological activity screening tests, the n-butanol fraction showed stronger antioxidant activities than the other four fractions and it was also the potent fraction for in vivo efficacy study of the protective effects against ulcerative colitis (Fig. S1).

Metabolomics based on high throughput sensitive UPLC-HESI-MSMS enabled in-depth studies on secondary metabolites in several parts from MA and revealed 76 different compounds, mainly phenolic, flavonoids and 12 different fatty acids. The individual compounds were identified via the exact molecular masses with ∆ less than 5 ppm, mass spectra and retention times and were compared with those of the standard compounds, as well as databases available online (PubChem, ChEBI, Metlin and KNApSAck) and literature data (Table 2).

Excessive production of cytokines as Ilβ6 lead to severe inflammation which can be suppressed by natural compounds as phenolics present in natural products like *p*-coumaric acid, rutin caffeic acid which inhibits induction of lipopolysaccharide inducible nitric oxide synthase production, also flavonoids as naringenin, quercetin prevent expression of inducible nitric oxide synthase protein through inhibition of nuclear factor-κB that represents the major transcripting factor for inducible nitric oxide synthase^41^.

In the current work, a comprehensive characterization of secondary metabolites using LC/MSMS was accomplished in the hydroalcoholic MA waste extract, as well as in the oil fraction identified by GC/MS. The analysis explained 75 secondary metabolites belonging to simple phenols, amino acids, phenolic acids, cinnamic acid derivatives and flavonoids in addition to sugars. Total flavonoid and phenolic contents were more pronounced in the butanol extract. The latter also exhibited potent anti-inflammatory bowel disease “ulcerative colitis” (Fig. S1).

Phenolic acids are aromatic carboxylic acid with hydroxyl derivatives that have only one phenolic ring in their structure. They include two types; hydroxybenzoic acid and hydroxycinnamic acid derivatives^42^. Caffeic, p-coumaric, ferulic and sinapic acids are the hydroxycinnamic acid derivatives that are more abundant in plants as compared to the benzoic acid derivatives; such as gallic acid, protocatechuic acid and p-hydroxybenzoic acid (Table 2).

### Pharmacological study

#### In vitro study

**Figure 3:**
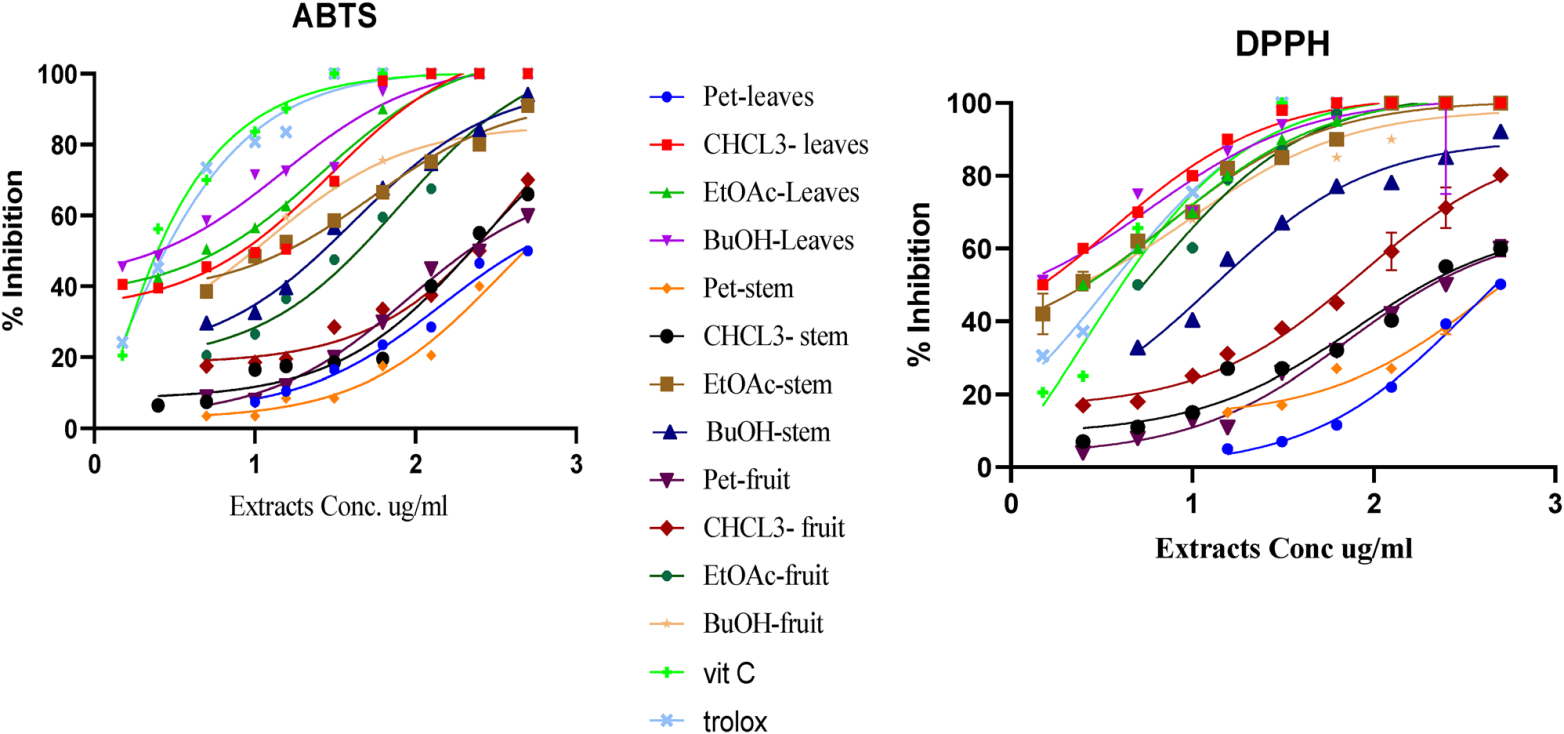
In vitro IC_50_ DPPH and ABTS antioxidant activity of different plant parts of *Musa acuminata* extracts.

Results of the present study revealed that the highest concentrations of IC_50_ in both DPPH and ABTS antioxidant were found in BuOH-Leaves; 5.85: 14.92, then BuOH-fruit peels; 9.94:12.08 and BuOH-pesudostem; 13.17:41.08, respectively) Fig. 3 and Table S2. These findings are in agreement with Oresanya et al*.*^43^.

#### Acute and sub chronic toxicity studies

In the present acute toxicity study MA leaves, pseudo-stem and fruit peels extracts given to three groups rats in a single dose of 5000 mg/kg; all were given once; exhibited no mortalities during the first twenty four hours after administration. The percentage of body weight change of the group that received pseudo-stem extract showed significant decrease while the group that received fruit peels extract showed significant increase compared to negative control group (Table S4). However there weren’t any changes in bowel habits, also there weren’t any changes in behaviour or hair loss or discolouration in all groups during the two successive weeks duration of the experiment.

Moreover histopathologic examination of both liver and kidneys revealed normal hepatic parenchyma and normal hepatocytes (Fig. 5a), and normal renal tubules and glomeruli (Fig. 7a).

Accordingly the selected doses for testing the sub-chronic toxicity of all extracts were 250 and 500 mg/kg given orally for fourteen successive days, which is the same duration of the efficacy study. Observation of rats for any marked change in body weights (Table S5), or gross bowel habit changes as severe or frequent motions or severe constipation revealed that they were the same as negative control group, also their behaviour was the same as negative control group.

Assessment of both liver and kidney functions in the subchronic toxicity study by measuring ALT, AST, Urea and Creatinine levels in sera of treated rats (Table 3), in the subchronic toxicity study showed non-significant variation from negative control group.

**Table 3:** Effect of oral administration of MA leaves, pseudostem and fruit peels extracts on liver and kidney function tests of rats in subchronic toxicity study.

Groups	Parameter			
	ALT (U/L)	AST (U/L)	Urea (mg/dL)	Creatinine (mg/dL)
Negative control	36.08 ± 2.54	27.78 ± 1.14	30.1 ± 1.74	0.95 ± 0.03
MA leaves (250 mg/kg)	34.9 ± 0.38	21.22 ± 0.9	27.52 ± 2.9	0.84 ± 0.05
MA leaves (500 mg/kg)	30.05 ± 0.31	24.32 ± 1.8	24.5 ± 3.84	0.79 ± 0.04
MA pseudostem (250 mg/kg)	40.97 ± 2.29	27.64 ± 2.96	36.49 ± 0.07	0.93 ± 0.03
MA pseudostem (500 mg/kg)	35.43 ± 3.29	22.51 ± 2.33	27.52 ± 0.78	0.71 ± 0.05
MA fruit peels (250 mg/kg)	41.9 ± 1.64	23.17 ± 1.7	33.15 ± 0.54	0.72 ± 0.1
MA fruit peels (500 mg/kg)	27.92 ± 0.72	26.78 ± 2.8	22.14 ± 1.87	0.78 ± 0.02

Results are expressed as means of levels of ALT, AST, urea and creatinine in rat sera ± SE. n = 8; Data were analysed using one way analysis of variance (ANOVA) followed by Tukey Kramer’s multiple comparison test. No significant difference detected among groups.

In the present study, results of both acute and subchronic toxicity studies denoted the safety of MA leaves, pseudostem and fruit peels to be used in the efficacy study as protective agents against inflammatory model of rat distal part of colon mimicking ulcerative colitis in humans.

#### Efficacy study

In the present study the efficacy of MA leaves, pseudo-stem and fruit peels extracts was evaluated as potential protective supplements against colonic inflammatory disease in a rat model mimicking ulcerative colitis in human patients.

Ulcerative colitis was induced by per rectal injection of 2 ml 8% acetic acid. Treatment with MA extracts was given orally to rats in doses of 250 and 500 mg/kg of each extract for 14 days prior to induction of ulcerative colitis. The doses were selected according to the results of toxicity studies formerly done in the present work.

The weights of all treated rats involved in the study were within normal and didn’t show any significant difference from the negative control group, also the % change of weights at the end of experiment compared to those before starting was minimal. The non significant change in body weights denotes that the extracts don’t alter normal bowel habits and don’t affect the appetite of rats as food consumption was constant throughout the experiment (Table S5).

It was noticed that untreated positive control rats suffered severe diarrhoea within the twenty four hours period following acetic acid per rectal infusion for induction of UC. This finding varied in intensity from mild to absent in all other groups, which denotes that acetic acid led to severe irritation.

Evaluation of the effects of pretreatments was performed by macroscopic examination of dissected colons by naked eyes (Table 4), and by histopathologic examination (Table 5) followed by immune-histochemical examination (Table 6), and finally biochemical assay for detection of inflammatory markers (Table 7), in addition to the qualitative test antineutrophil cytoplasmic antibodies(ANCA) which is specific for UC detection.

**Table 4:** Macroscopic grading of the protective effect of oral administration of MA leaves, pseudostem and *fruit peels* extracts on colonic ulceration.

Groups	Ulcer grading			
	Number	Severity	Ulcer index	% of protection
Negative control	–	–	–	–
Positive control Acetic acid 8% (2 ml/rat)	5	5	1100	–
Prednisolone (5 mg /kg)	1	0.5	640 ± 4.2^@^	41.81 ± 0.38
MA leaves (250 mg/kg)	3.25	2.62	933.75 ± 10 76^@^*	15.11 ± 0.97*
MA leaves (500 mg/kg)	2	1.37	788.57 ± 8.85^@^*^#^	28.31 ± 0.8*^#^
MA pseudostem (250 mg/kg)	2	1.62	786.25 ± 8.64^@^*^#^	28.52 ± 0.78*^#^
MA pseudostem (500 mg/kg)	1.87	1.25	656.25 ± 9.89^@#$&^	40.34 ± 0.89^#$&^
MA fruit peels (250 mg/kg)	1.37	0.87	647.5 ± 7.5^@#$&^	41.13 ± 0.68^#$&^
MA fruit peels (500 mg/kg)	0.77	0.55	513.33 + 5.59^@^*^#$!€^	53.33 ± 0.38*^#$&!€^

Results are expressed as means of ulcer number, severity, index and % of treatment protective effect ± SE after 14 days of MA extract and prednisolone administration. followed by ulcer induction by single pr acetic acid infusion. n = 8; Data were analysed using one way analysis of variance (ANOVA) followed by Tukey Kramer’s multiple comparison test; Significance was considered at P ≤ 0.05.

^@^Significantly different from positive control group, *significantly different from prednisolone group, ^#^significantly different from MA leaves 250 mg/kg group, ^$^significantly different from MA leaves 500 mg/kg group, ^&^significantly different from MA pseudostem 250 mg/kg group, ^!^significantly different from MA pseudostem 500 mg/kg group, ^€^significantly different from MA *fruit peels* 250 mg/kg group.

**Table 5:** Pathologic scoring of colon tissue damage assessed in the normal and treated groups.

Group	Histopathologic lesion scoringng (mean ± SD)
Normal	0.10 ± 0.31
Positive control (C+ ve) acetic acid 8% (2 ml/rat)	5.00^a^ ± 0.00
Prednisolone	1.00^d^ ± 0.94
Musa leaves (250 mg/kg)	4.80^a^ ± 0.42
Musa leaves (500 mg/kg)	3.20^b^ ± 0.78
Musa pseudostem (250 mg/kg)	2.30^c^ ± 0.82
Musa pseudostem (500 mg/kg)	1.90^b,c^ ± 1.28
Musa fruits (250 mg/kg)	1.50^c,d^ ± 1.17
Musa fruits (500 mg/kg)	0.80^d,e^ ± 0.63

**Table 6:** MPO expression recorded in the colonic mucosa and submucosa of normal and treated groups.

Group	MPO expression in the colonic mucosa (% of positive cells/HPF) (mean ± SD)	MPO expression in the colonic mucosa (% of positive cells/HPF) (mean ± SE)	MPO expression in the colonic submucosa (% of positive cells/HPF) (mean ± SD)	MPO expression in the colonic submucosa (% of positive cells/HPF) (mean ± SE)
Negative control	0.20^d^± 0.42	0.20^d^ ± 0.13	0.10^d^ ± 0.31	0.10^d^ ± 0.10
C+ ve acetic acid 8% (2 ml/rat)	3.30^a^ ± 0.48	3.30^a^ ± 0.15	3.90^a^ ± 0.31	3.90^a^ ± 0.10
Prednisolone	0.80^c,d^ ± 0.63	0.80^c,d^ ± 0.20	0.80^c,d^ ± 0.42	0.80^c,d^ ± 0.13
Musa leaves (250 mg/kg)	3.30^a^ ± 0.67	3.30^a^ ± 0.21	3.40^b^ ± 0.69	3.40^b^ ± 0.22
Musa leaves (500 mg/kg)	2.20^b^ ± 1.13	2.20^b^ ± 0.35	1.30^c^ ± 0.67	1.30^c^ ± 0.21
Musa pseudostem (250 mg/kg)	1.10^c^ ± 0.73	1.10^c^ ± 0.23	1.10^b,c^ ± 0.73	1.10^b,c^ ± 0.29
Musa pseudostem (500 mg/kg)	0.90^c^ ± 0.56	0.90^c^ ± 0.17	0.70^c,d^ ± 0.40	0.70^c,d^ ± 0.15
Musa fruits (250 mg/kg)	1.00^c^ ± 0.47	1.00^c^ ± 0.14	1.00^b,c^ ± 0.66	1.00^b,c^ ± 0.21
Musa fruits (500 mg/kg)	0.50^c,d^ ± 0.52	0.50^c,d^ ± 0.16	0.20^d^ ± 0.42	0.20^d^ ± 0.13

Results are expressed as means of MPO positive cells ± SE.

^a^Significantly different from negative control group, ^b^Significantly different from prednisolone group, ^c^Significantly different from Musa leaves high dose, d = Significantly different from positive control group.

**Table 7:** CRP and ILβ6 anti-inflammatory activity of MA leaves, pseudo-stem and fruit peels.

Group (parameter)	CRP (ng/ml)	ILβ6 (pg/ml)
Negative control	1.36 ± 0.08	43.83 ± 1.86
Positive control group acetic acid 8% (2 ml/rat)	3.07 ± 0.11^@^	114.3 ± 2.62^@^
Reference group prednisolone (5 mg/kg)	2.18 ± 0.01^@^*	54.5 ± 0.51*
MA leaves extract (250 mg/kg)	2.64 ± 0.04^@$^	80.58 ± 5.37^@^*^$^
MA leaves extract (500 mg/kg)	2.61 ± 0.13^@^	69.43 ± 2.82^@^*
MA pseudostem extract (250 mg/kg)	2.44 ± 0.23^@^*	70.3 ± 3.58^@^*^$^
MA pseudostem extract (500 mg/kg)	2.15 ± 0.17^@^*	63.7 ± 2.16^@^*
MA fruit peels extract (250 mg/kg)	2.57 ± 0.13^@^	60.57 ± 0.99^@^*
MA fruit peels extract (500 mg/kg)	1.88 ± 0.16*	56.78 ± 2.75*

Results are expressed as mean of levels of CRP and IL β6 ± S.E in serum of rats treated with MA leaves ,pseudo-stem and *fruit peels* (250 and 500 mg/kg) and Prednisolone (5 mg /kg) for two successive weeks followed by induction of colon lesions by using acetic acid 8%(2 ml/rat); n = 8; Data were analysed using one way analysis of variance (ANOVA) followed by Tukey Kramer’s multiple comparisons test; Significant at P ≤ 0.0001.

^@^Significant different from negative control; *Significant difference from positive control group; ^$^Significant difference from prednisolone group.

**Figure 4:**
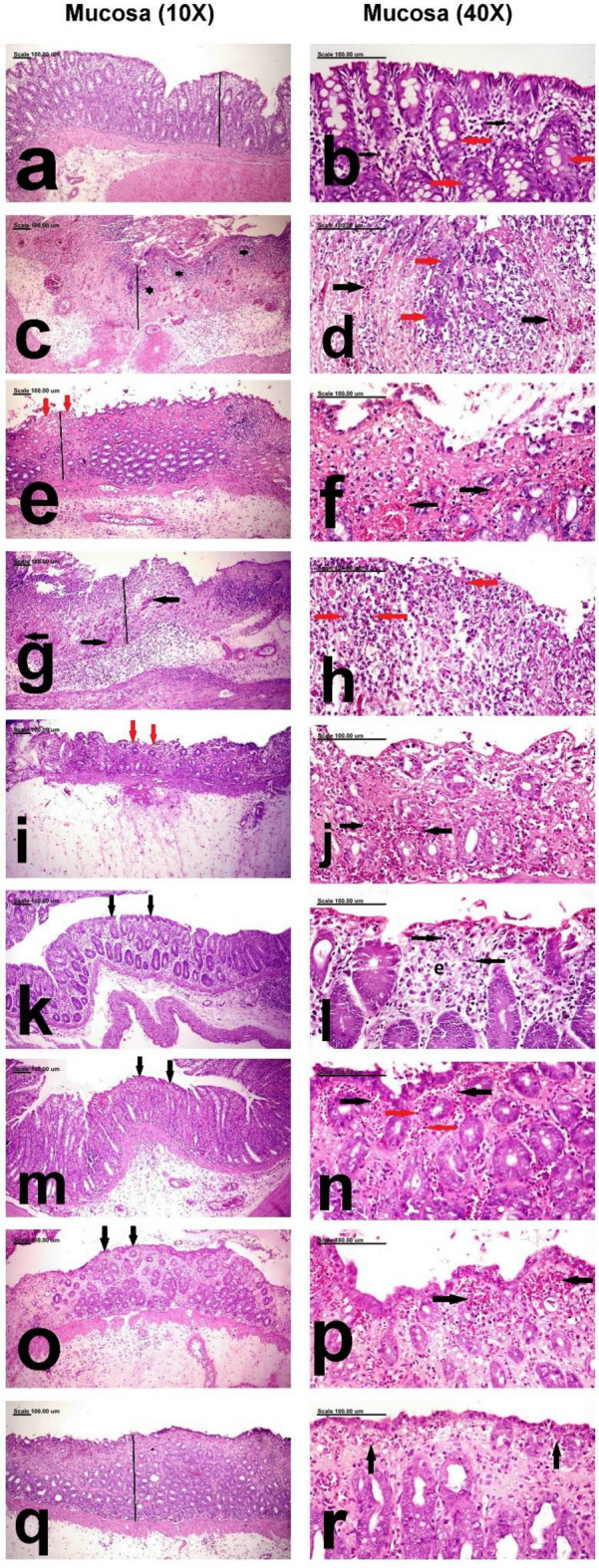
Photomicrograph showing the ulcerative colonic mucosa photomicrograph from the colon mucosa of, (a,b) negative control rats showing normal mucosa (black line) (**a**) and normal lamina propria containing few eosinophils (black thin arrows) as well as normal crypts lined by mucin-secreting cells (red thick arrows) (**b**), (**c**,**d**) C+ ve showing diffuse ulcerative colitis with diffuse necrosis and desquamation of mucosal epithelium (black line) and crypts which are intensely infiltrated by neutrophils (astrix) (**c**) in addition to severe congestion of mucosal blood vessels (black arrows) and aggregation of bacterial colonies (red arrows) (**d**), (**e**,**f**) Prednisolone treated group showing small focal ulcerative lesions (black line) with focal necrosis and desquamation of mucosal epithelium (red arrows) (**e**) and few proprial hemorrhage (black arrows) (**f**), (**g**,**h**) Leave (250 mg/kg)group showing diffuse necrosis of colonic mucosa(black line) associated with severe congestion of mucosal blood vessels(black arrows) (**g**) and massive neutrophilic cell infiltration (red arrows) (**h**), (**i**,**j**) the Leave (500 mg/kg)group showing large focal erosive lesion (red arrows) (**i**) and few proprial hemorrhage (black arrows) (**j**), (**k**,**l**) Stem (250 mg/kg) group showing normal mucosal epithelium (black arrows) (**k**) and mild proprial edema (**e**) as well as few leukocytic cell infiltration (black arrows), (**m**,**n**) Stem (500 mg/kg) group showing regeneration of the mucosal epithelium (black arrows) (**m**) and minimal leukocytic cell infiltration (red arrows) as well as scant proprial hemorrhage (black arrows) (**n**), (**o**,**p**) Fruit peels (250 mg/kg) treated group showing regeneration of mucosal epithelium (black arrows) (**o**) and scant proprial hemorrhage(black arrows) (**p**), and (**q**,**r**) Fruit peels (500 mg/kg) treated groups showing normal colonic mucosa (black line) (**q**) and scant proprial hemorrhage (black arrows) (**r**). (Stain:H&E; Scale bar = 100 µm).

**Figure 5:**
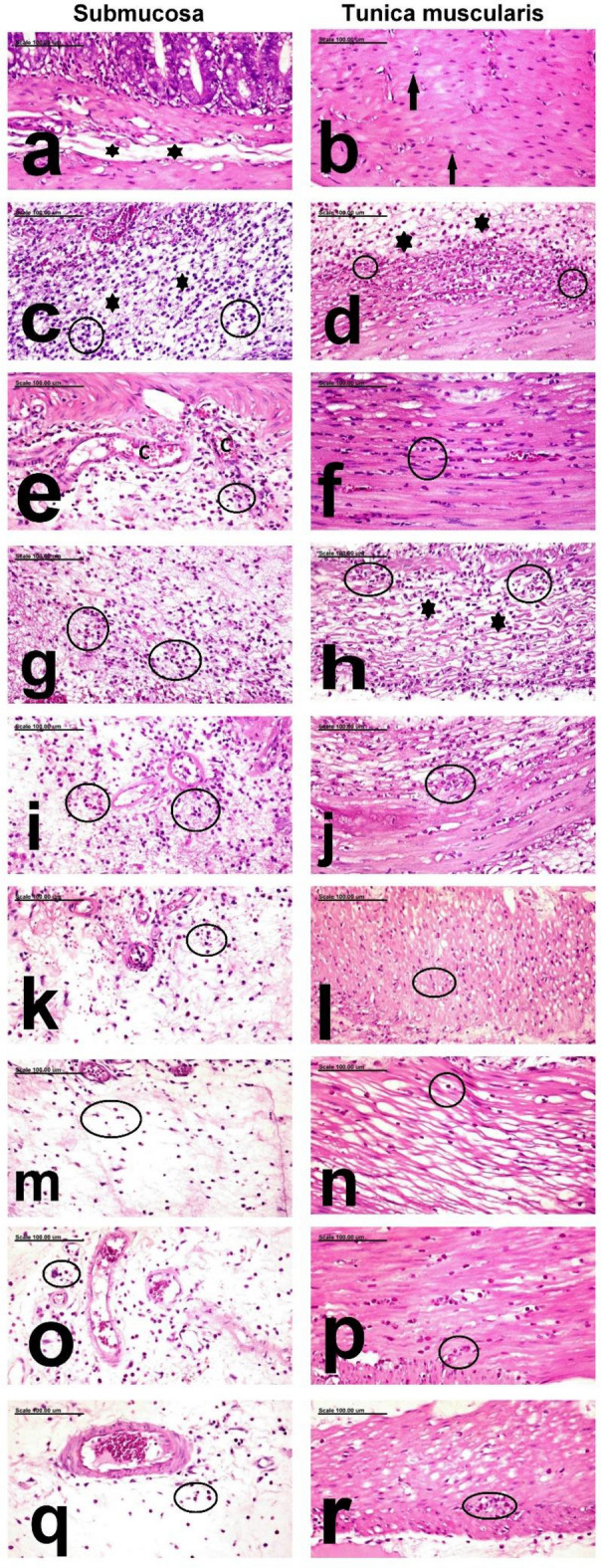
Photomicrograph showing the edematous and inflammatory reaction of the colonic submucosa and musculosa photomicrograph from the colon submucosa and T.muscularis of, (**a**,**b**) negative control rats showing normal submucosa (astrix) (**a**) and T.muscularis (black arrows) (**b**), (**c**,**d**) C+ ve showing expansion of the submucosa (**c**) and T.muscularis (**d**) by edematous fluids (astrix) and intense neutrophilic cell infiltration (black circle), (**e**,**f**) Prednisolone treated group showing congestion of the submucosal blood vessels (C) and infiltration of the submucosa (**e**) and T.muscularis (**f**) with few neutrophils (black circle), (**g**,**h**) Leave (250 mg/kg)group showing intense infiltration of the submucosa with neutrophils (black circles) (**g**) and marked separation of muscle fibers of T.muscularis by edematous fluid (astrix) and leukocytic cell infiltration (black circles) (**h**), (**i**,**j**) the Leave (500 mg/kg)group showing intense infiltration of the sub mucosa (**i**) and T.muscularis (**j**) with neutrophils (black circles), (**k**,**l**) Stem (250 mg/kg) group showing mild infiltration of submucosa (**k**) and T.muscularis (**l**) with neutrophils (black circles), (**m**,**n**) Stem (500 mg/kg) treated group showing few neutrophils (black circles) infiltrating the submucosa (**m**) and T.muscuaris (**n**), (**o**,**p**) Fruit peels(250 mg/kg) treated group showing mild infiltration of the submucosa (**o**) and T.muscularis (**p**) with neutrophils (black circles), and (**q**,**r**) Fruit peels(500 mg/kg) treated group showing Sparse neutrophils (black circles) in the submucosa (**q**) and T.muscularis (**r**). (Stain:H&E; Scale bar = 100 µm).

Macroscopic examination of colons dissected from negative control group showed intact mucosa with no signs of inflammation or haemorrhagic spots (score 0). Microscopic examination of mucosa of colons of rats in this group was normal and the lamina propria was normal with few eosinophils and normal crypts that were lined by mucin-secreting cells **(**Fig. 4a,b), and both submucosa and T-muscularis (Fig. 5a,b) were also normal which was consistent with gross examination of negative control colons.

**Figure 6:**
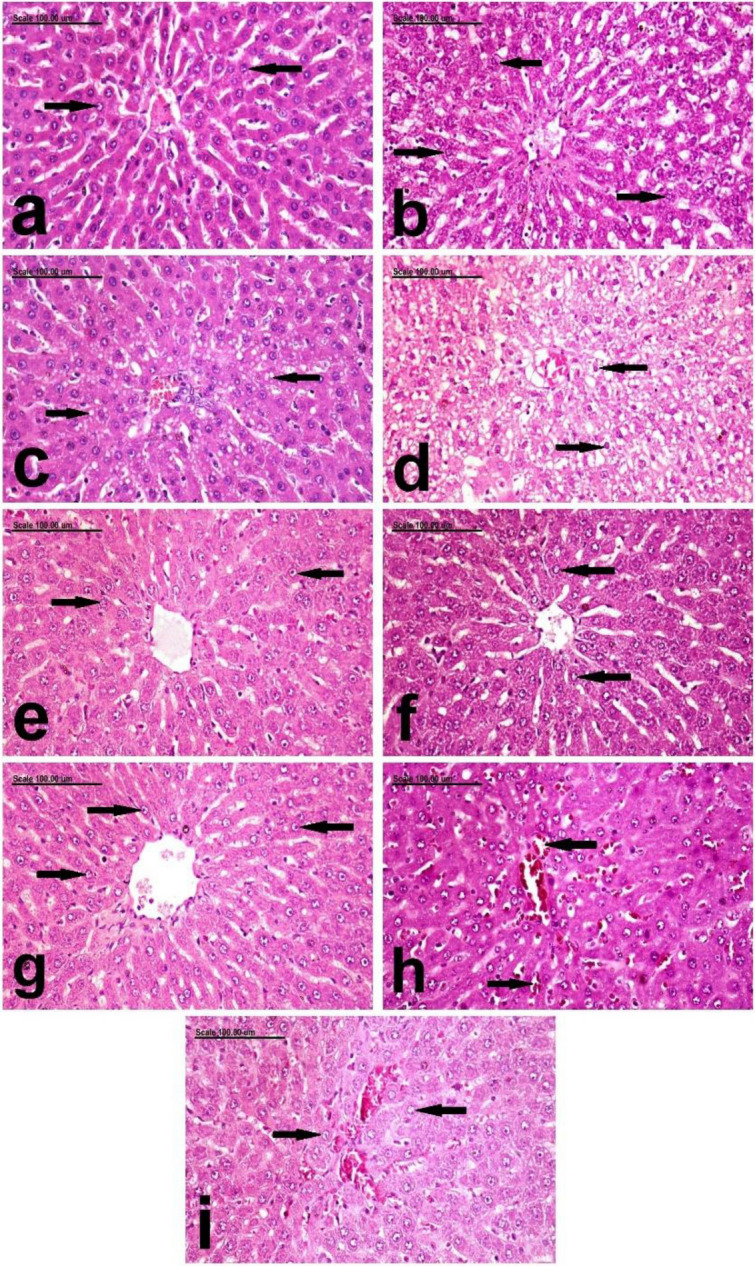
Photomicrograph showing the pathological alterations of the hepatic tissue. photomicrograph from the liver of, (**a**) negative control rats showing normal hepatic parenchyma with normal hepatocytes (arrows), (**b**) C+ ve showing mild granular degeneration of hepatocytes (arrows), (**c**) Prednisolone treated group showing mild focal vacuolar degeneration of hepatocytes (arrows), (**d**) the low dose leave group showing swelling and vacuolation of hepatocellular cytoplasm (arrows), (**e**) the high dose leave group showing normal hepatocytes (arrows), (**f**) low stem-treated groups showing normal histological structures (arrows), (**g**) high dose stem-treated groups showing normal hepatocytes (arrows), (**h**) low dose fruit peels treated group showing mild focal congestion of some hepatic sinusoids (arrows), and (**i**) high dose fruit peels treated groups showing normal heptic parenchyma (arrows) (Stain: H&E; Scale bar = 100 µm).

**Figure 7:**
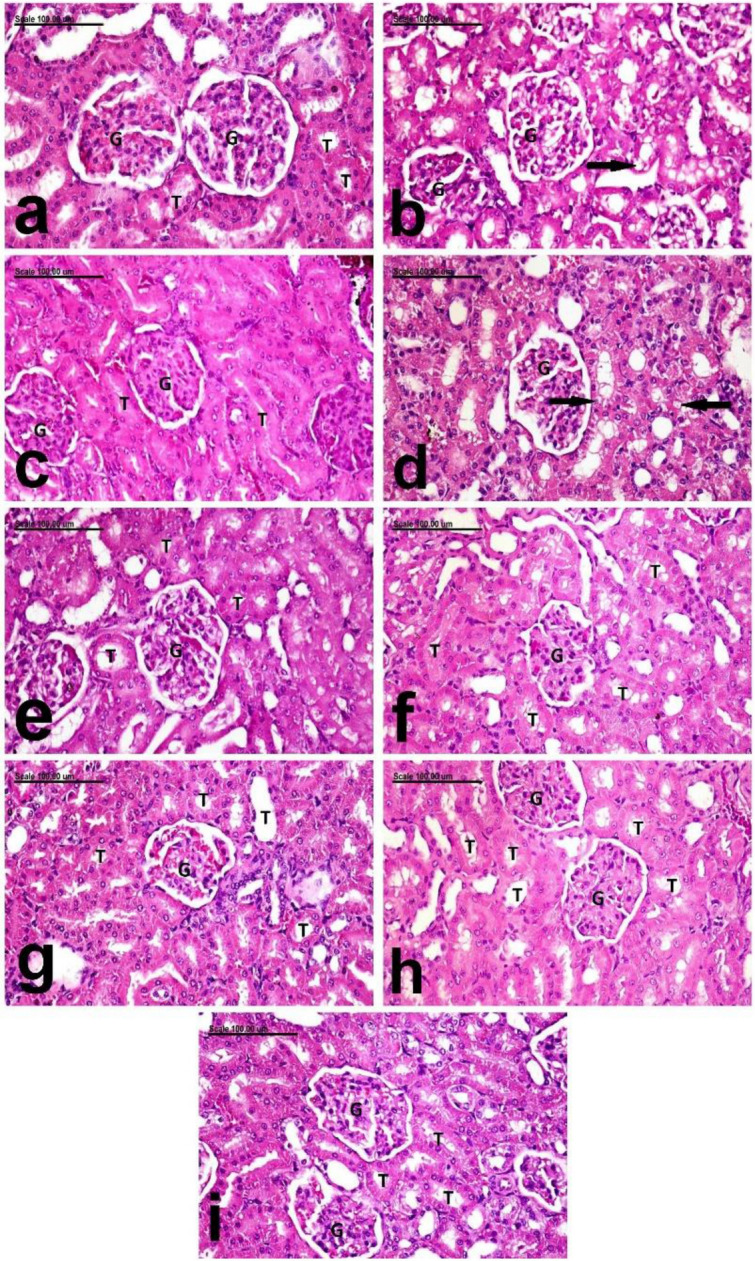
Photomicrograph showing the pathological alterations of the renal tissue. photomicrograph from the kidneys of, (**a**) negative control rats showing normal renal tubules (T) and glomeruli (G), (**b**) C+ ve showing vacuolation of individual cells (arrow) lining the renal tubules, (**c**) Prednisolone treated group showing normal renal tubules (T), (**d**) the low dose leave group showing vacuolization of some renal tubular epithelial cells (arrows), (**e**) the high dose leave group showing normal renal tubules (T), (**f**) low stem-treated groups showing normal histological structures, (**g**) high dose stem-treated groups showing normal renal parenchyma, (**h**) low dose fruit peels treated group showing normal histological structures, and (**i**) high dose fruit peels treated groups showing normal renal parenchyma. (Stain: H&E; Scale bar = 100 µm).

On the other hand colons dissected from untreated rats that received only acetic acid per rectum were severely ulcerated to the degree of perforation with grossly detected haemorrhagic areas in 100% of rats, which was also confirmed by histopathologic examination as severe deleterious histopathological lesions were demonstrated in the colon of Positive Control (C+ ve) group, with increased pathologic lesion scoring. These histopathological lesions were characterized by diffuse ulcerative colitis with diffuse necrosis and desquamation of mucosal epithelium and complete necrosis as well as fragmentation of the crypts which are intensely infiltrated by neutrophils in addition to severe congestion of mucosal blood vessels (Fig. 4c) in addition to aggregation of bacterial colonies (Fig. 4d). The submucosa and tunica muscularis are greatly expanded by edematous fluids and neutrophilic cell infiltration (Fig. 5c,d, respectively). Liver showed mild granular degeneration of hepatocytes (Fig. 6b), in comparison to the negative control group, which showed normal hepatic structure (Fig. 6a). Vacuolation of individual cells lining the renal tubules were demonstrated in the kidneys of this group (Fig. 7b).

On the other hand macroscopic examination of group treated with prednisolone which was used as a standard drug revealed significant reduction in ulcer index as 62.5% of rats were affected and showed significant increase in percentage of ability of protection against UC (41.81%) compared to untreated group,the results were consistent with histopathology, which revealed pronounced improvement with significant decrease of pathologic lesion scoring, which revealed small multifocal ulcerative lesions with focal necrosis and desquamation of mucosal epithelium, focal mononuclear inflammatory cell infiltration (Fig. 4e) and few proprial hemorrhage (Fig. 4f). The submucosa and T.muscularis are infiltrated by few neutrophils (Fig. 5e,f, respectively). Mild focal vacuolar degeneration of hepatocytes was demonstrated in the liver (Fig. 6c), but normal renal tubules were demonstrated in the kidneys (Fig. 7c).

In contrast to Prednisolone, gross examination by naked eye of group treated with leaves 250 mg/kg revealed increased number and severity of ulcers in 87.5% of pretreated rats, with no significant improvement where the ability of protection against ulceration was only 15.1%, and that was confirmed by histopathology as there was diffuse necrosis of colonic mucosa associated with severe congestion of mucosal blood vessels and massive neutrophilic cell infiltration were frequently observed (Fig. 4g,h).In addition, intense infiltration of the submucosa with neutrophils was marked (Fig. 5g). The T.muscularis revealed marked separation of muscle fibers by edematous fluid and leukocytic cell infiltration (Fig. 5h). Swelling and vacuolation of hepatocellular cytoplasm were demonstrated in the liver (Fig. 6d). In addition, vacuolization of some renal tubular epithelial cells were demonstrated in the kidneys (Fig. 7d).

In comparison to low dose leave group, significant amelioration was recorded in the high dose leave group (500 mg/kg), by gross examination as the ulcer index was significantly reduced and the percent of protection of the high dose extract was 28.31% which was significantly higher than both low leave extract and positive control group as ulceration was detected only in 75% of pretreated rats, yet it was significantly less than prednisolone group. Consistently histopathologic examination revealed large focal erosive lesion and few proprial hemorrhage (Fig. 4i,j). But the sub mucosa and T.muscularis were intensely infiltrated with neutrophils (Fig. 5i,j, respectively). The liver and kidneys of this group appeared normal (Figs. 6e and 7e, respectively).

The group pretreated with pseudostem 250 mg/kg showed better macroscopic examination profile as the number and severity of ulcers was less as they were detected in only 75% of pretreated rats which consequently significantly reduced the ulcer index compared to leaves pretreated group by low and dose and also to positive control group, but its protective effect was significantly less than prednisolone and almost the same as leaves pretreated group with high dose as the % of protection was 28.52% in pseudostem low dose group. The histopathologic examination of pseudostem low dose revealed pronounced attenuation of the pathological lesions with decreases pathologic lesion scoring,small focal necrosis of mucosal epithelium associated with mild proprial edema and few leukocytic cell infiltration were demonstrated (Fig. 4k,l). Mild infiltration of submucosa and T.muscularis with neutrophils was recorded in this group (Fig. 5k,l, respectively). Normal histological structures of liver and kidneys were also demonstrated (Figs. 6f and 7f, respectively).

Regarding the gross examination of the high dose of pseudostem (500 mg/kg) and low dose of fruit peels extract (250 mg/kg), ulcers were detected in only 62.5%, which is the same percentage of affected rats in the prednisolone (standard),also the number and severity of ulcers were approximately close to each other leading to non significant differences in ulcer indices and consequently % of protection of both pseudostem extract high dose (40.34%) and fruit peels extract low dose (41.13%)on one side, and prednisolone (41.81%) which is the standard treatment on the other side, however their protective effects were significantly higher than those of leaves low and high doses as well as pseudostem low dose, and of course the positive control group. On the other hand they showed significant lower protective effects than fruit peels extract high dose (500 mg/kg), whose protective effect was the highest of all pretreatments when compared to positive group and each pretreatment with pronounced significant % of protection of 53.33% and least number of ulcers, severity and affection of only 50% of rats, consequently exhibiting the least ulcer index among all other groups. The histopathologic photomicrographic findings were consistent with the macroscopic examination of these groups, where normal colonic mucosa in most examined sections was frequently demonstrated in high dose stem-treated groups stem group. Regenerative activity of the mucosal epithelium and minimal leukocytic cell infiltration as well as scant proprial hemorrhage were demonstrated in high dose stem-treated groups (Fig. 4m,n). The submucosa was infiltrated with few neutrophils (Fig. 5m) and the T.muscuaris showed edema with few neutrophilic cell infiltration (Fig. 5n). Normal hepatocytes and renal parenchymal structures were also demonstrated (Figs. 6g and 7g, respectively). Much better improvement, with marked regenerative activity of the colon mucosa and proliferation of colonic lymphoid nodules and minimal leukocytic cell infiltration were recorded in the mucosa of low dose fruit peels treated groups (Fig. 4o,p) and high dose fruit peels treated groups (Fig. 4q,r). The submucosa and T.muscularis of low dose fruit peels treated groups were mildly infiltrated with neutrophils (Fig. 5o,p). Only mild focal congestion of some hepatic sinusoids were demonstrated in this group (Fig. 6h), but normal histological structures were demonstrated in the kidneys (Fig. 7h) Sparse neutrophils were demonstrated in the submucosa and T.muscularis of high dose fruit peels treated groups (Fig. 5q,r). Normal heptic and renal parenchyma were demonstrated (Figs. 6i and 7i, respectively).

One of the most important immune-histochemical diagnostic criteria of UC, is the excessive detection of neutrophil cytoplasmic primary granules that are loaded with MPO inflammatory enzyme. When the severity of UC increases, the activity of MPO increases due to increase its release from increased activated immune cells as neutrophils and macrophages^44^. It is reported that Neutrophil-MPO augments inflammation and tissue damage with excessive production of free radicals^45^.

**Figure 8:**
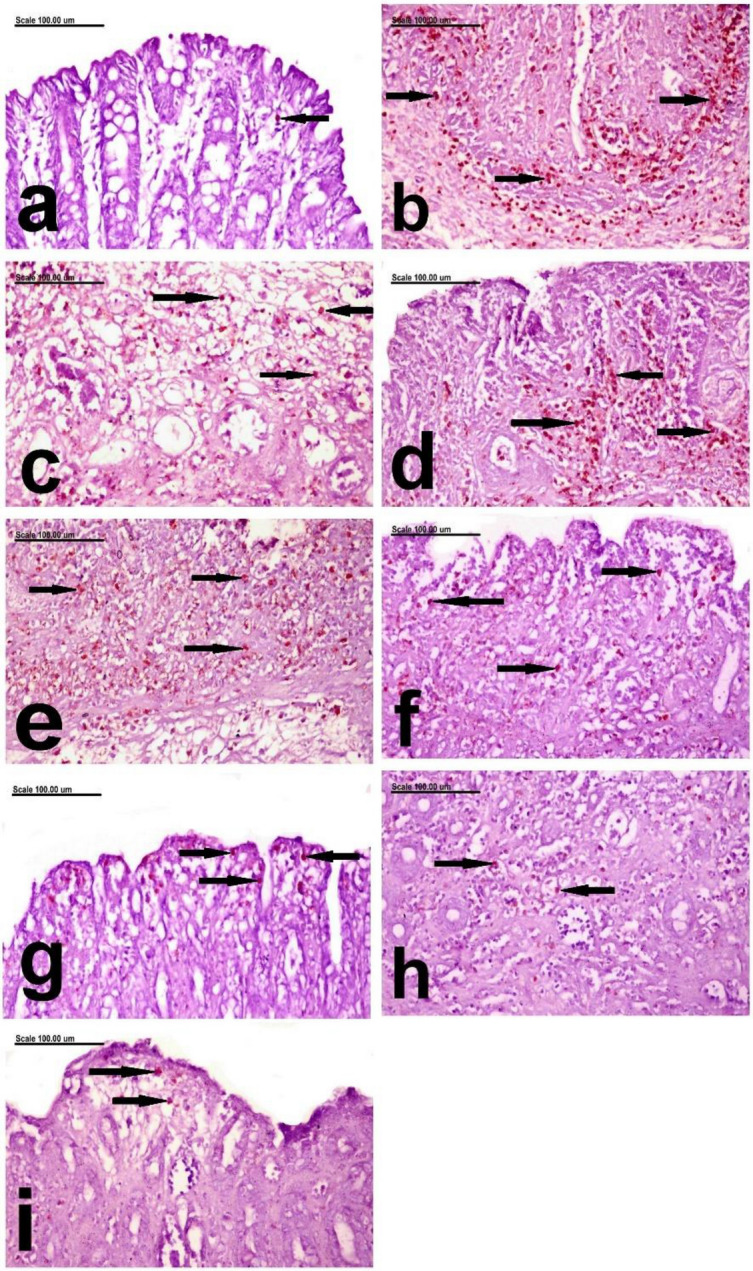
Photomicrograph showing the distribution of MPO-positive cells in the colonic mucosa photomicrograph from the MPO immunohistochemically-colon mucosa of, (**a**) negative control rats showing Individual MPO+ cells in the mucosa (arrow), (**b**) C+ ve group showing increase of % of MPO+ cells (arrows) in the colonic mucosa, (**c**) Prednisolone treated group showing significant decrease of % of MPO+ cells (arrows), (**d**) the low dose leave group showing increased % of MPO+ cells (arrows), (**e**) the high dose leave group showing decreased % of MPO+ cells (arrows), (**f**) low stem-treated groups showing marked decrease of % of MPO+ cells (arrows), (**g**) high dose stem-treated groups showing significant decrease of % of MPO+ cells (arrows), (**h**) low dose fruit peels treated group showing remarkable decrease of MPO+ cells (arrows), and (**i**) high dose fruit peels treated groups showing few scattered MPO+ cells (arrows).(MPO immunohistochemical staining; Scale bar = 100 µm).

**Figure 9:**
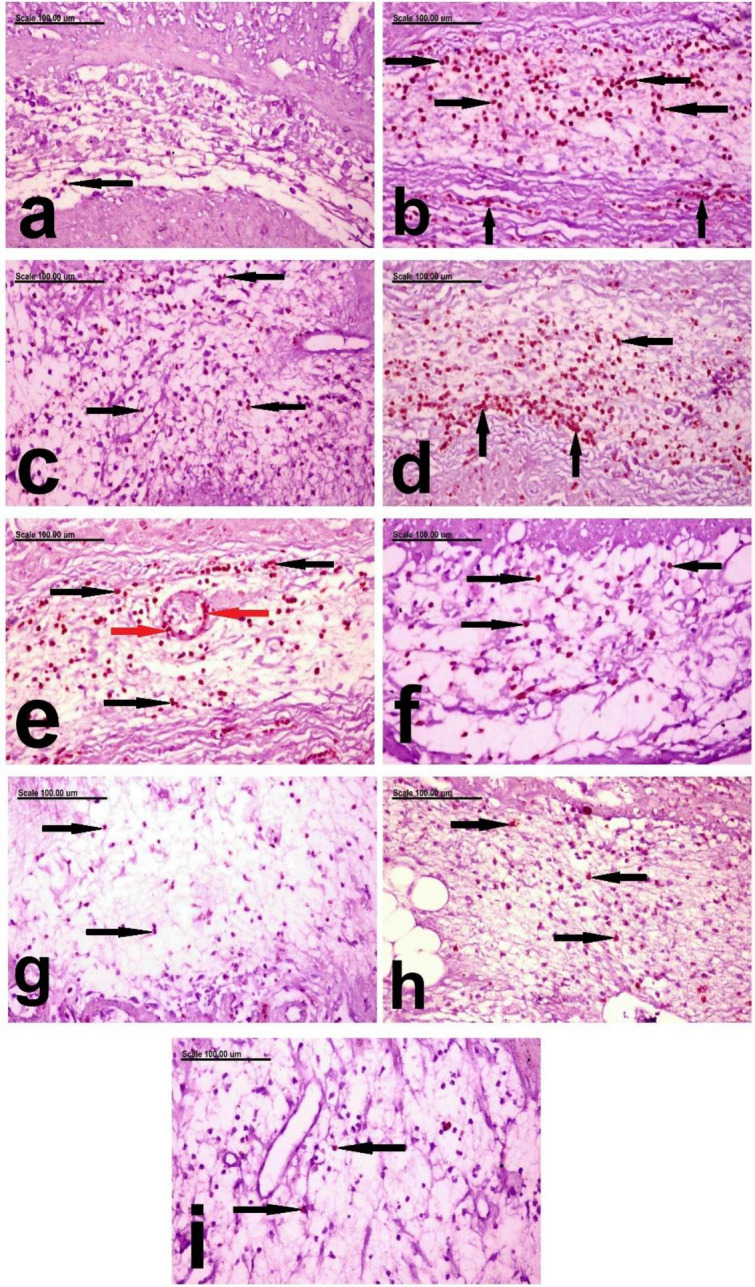
Photomicrograph showing the distribution of MPO-positive cells in the colonic sub-mucosa photomicrograph from the MPO immunohistochemically-colon submucosa of, (**a**) negative control rats showing Individual brown stained MPO+ cells (arrow) in the mucosa, (**b**) C+ ve rats showing numerous intensely brown stained MPO+ cells in the colonic submucosa and T.muscularis (arrows), (**c**) Prednisolone treated group showing significant decrease of brown stained MPO+ cells (arrows), (**d**) the low dose leave group showing abundant brown stained MPO+ cells (arrows), (**e**) the high dose leave group showing brown stained MPO+ cells in the wall of the submucosal blood vessel (red arrows) and the submucosa (black arrows), (**f**) low stem-treated groups showing marked decrease of % of MPO+ cells (arrows), (**g**) high dose stem-treated groups showing remarkable decrease of % of MPO+ cells (arrows), (**h**) low dose fruit peels treated group showing significant decrease of MPO+ cells with brown staining (arrows), and (**i**) high dose fruit peels treated groups showing few scattered MPO+ cells (arrows). (MPO immunohistochemical staining; Scale bar = 100 µm).

In the present study, the results of *MPO immune-histochemical expression* recorded in the colonic mucosa and submucosa showed that individual MPO+ cells were demonstrated in the mucosa and submucosa of the colon of normal rats (Figs. 8a and 9a). Whereas, increased expression of MPO with significant increase of % of MPOpositively stained cells, with strong brown staining, was recorded in group given acetic acid infusion per rectum without prior treatment (Figs. 8b and 9b).This confirms the obtained results of histopathology, in which intense inflammatory cellular infiltrating the colonic mucosa and sub-mucosa was demonstrated. Significant decrease of % of MPO+ cells was recorded in the mucosa and submucosa of Prednisolone-treated group (Figs. 8c and 9c). The colon of low dose leave group showed increased % of MPO+ cells, which are insignificantly different from the C+ ve group, in the mucosa and submucosa (Figs. 8d and 9d). But significant difference was recorded in the high dose leave group in both the mucosa and submucosa (Figs. 8e and 9e). Better improvement with marked decrease of % of MPO+ cells was recorded in the mucosa (Fig. 8f,g) and submucosa (Fig. 9f,g) of low and high dose stem-treated groups, with insignificant difference between them. On the other hand, significant difference was recorded between low and high dose fruit peels treated groups. Remarkable decrease of MPO expression with significant decrease of MPO+ was recorded in the mucosa and submucosa of low dose fruit peels treated groups (Fig. 8h). Only few scattered MPO+ cells were demonstrated in the mucosa and submucosa of high dose fruit peels treated groups (Figs. 8i and 9i), while low dose fruit peels treated group showed significant decrease of MPO+ cells with brown staining (Fig. 9h).

Biochemical analysis of sera obtained from rats infused per rectally with acetic acid in this study aiming at inducing a rat model of UC, revealed highly significant elevation of inflammatory markers CRP and Ilβ6 in the untreated group positive control compared negative control and to all other treated groups. The degree of inflammation was variable between the treated groups, all showed significant protection but the highest were those of the groups given prednisolone and fruit peels extract in high dose as these groups showed non significant difference from the negative control group.

Regarding ANCA test which is a highly specific qualitative test for diagnosis of UC, it revealed 100% negativity in the negative control group and in all treated groups except leaves low dose where it was 75% negative. On the contrary, the positive control results were 100% positive. This finding is in enforced by Pang et al.^46^, in their study as they stated that ANCA test is diagnostic for UC and its quantification reveals the severity of UC^46^. These anti-inflammatory effects of MA led to protection against UC and owe to the high antioxidant capacity of the extracts of MA, which were investigated in this study. It was proven that there is increased incidence of digestive system ulceration in cases of increased oxidative stress, and on the other hand the ulcerogenic potential of some chemicals depletes with intake of sufficient antioxidant rich diet. This was explained by reduction of malondialdehyde and increased glutathione proportions in digestive system tissues^31^.

The anti-inflammatory effects of phenolics present in MA extract when they were given orally in our study are due to serial enzymatic reactions that take place in the digestive system. After being absorbed in the small intestine they conjugate with glucuronic acid and sulfonate, then 5–10% pass to the plasma^47^, but the largest portion (90–95%) pass directly to the large intestine (colon)^48^. where fermentation occurs by colonic microbiota, leading to elaboration of the positive effect of phenolics on colon’s health by reducing its pH as anti-inflammatory effects^47,49^ which was emphasized in our study and consequently suppression of cancer cells.

## Conclusion

In the present study an animal model mimicking ulcerative colitis was induced by using per rectal acetic acid infusion, it produced severe inflammation which could be prohibited by pretreatment with natural plant extracts rich in Flavonoids and phenolic acids due to their protective effects on the colon as 90–95% of phenolic acids are metabolized in the colon by microorganisms that lead to their anti-inflammatory activity.

This was clear in our study as characterization of secondary metabolites in MA revealed high contents of these compounds.

It is noteworthy mentioning that the MA fruit peels had the best effect regarding the ability to protect against development of severe ulcerative colitis, which introduces a promising natural supplement that can be used in future clinical studies for further evaluation of its effect as a protective pretreatment in vulnerable patients that are susceptible to have ulcerative colitis.

## Supplementary Information

Supplementary Information.

## Data Availability

Raw data of the present phytochemical and efficacy study are available in the supplementary file.

## Abbreviations

ABTS: 2,2′-Azino-bis(3-ethylbenzothiazoline-6-sulfonic acid)
ANCA: Anti-neutrophil cytoplasmic antibodies
CID: Collision-induced dissociation
CRP: C-reactive protein
C+ ve: Positive control group
DPPH: 2,2-Diphenyl-1-picrylhydrazyl
EI: Electron ionization
ESI: Electrospray ionization
FCR: Folin-Ciocalteu reagent
GCMS: Gas chromatography–mass spectrometry
HESI: Heated electrospray ionization
HPLC: High performance liquid chromatography
Gf: Gramm force
IL*β*6: Interleukin beta six
LCMSMS: Liquid chromatography–mass spectrometry
MA: *Musa acuminata*
MPO: Myeloperoxidase
RCF: Relative centrifugal force
Rpm: Rotations per minute
T.muscularis: Tunica muscularis
UC: Ulcerative colitis
UPLC: Ultra performance liquid chromatography
UPLC–QToF–MS: Ultra-high performance liquid chromatography-quadrupole time-of-flight mass spectrometry
