# Supplementary material for: Pharmacological and metabolomic profiles of Musa acuminata wastes as a new potential source of anti-ulcerative colitis agents
Source: Sci Rep. 2022 Jun 22;12:10595. doi: 10.1038/s41598-022-14599-8 (PMC9218116; doi:10.1038/s41598-022-14599-8)
Supplement: Supplementary file 1 — Supplementary Information. [file 41598_2022_14599_MOESM1_ESM.docx]

**Pharmacological and metabolomic profiles of *Musa acuminata* wastes as a new potential source of anti-ulcerative colitis agents**

Mona A. Mohammed^1#*^, Bassant M.M.Ibrahim^2#*^, Yasmin Abdel-Latif ^3&4^,Azza H.Hassan ^5^ , Mohamed A. El Raey^6^, Emad M. Hassan^1^, Souad E. El-Gengaihi^1*^.

^1^Medicinal and Aromatic Plants Research Department, Pharmaceutical Industries Research Division, National Research Centre, Giza, Egypt. Affiliation ID: 60014618.

^2^Pharmacology Department, Medical Research Division, National Research Centre, Dokki, Giza, Egypt, Po 12622, Affiliation ID: 60014618.

^3^ Medical Biochemistry Department, National Research Centre, Cairo, Egypt. PO 12622. Affiliation ID: 60014618.

^4^Faculty of Biotechnology, October University for Modern Sciences and Arts, 6th October, Giza, Egypt.

^5^Pathology Department, Faculty of Veterinary Medicine, Cairo University.

^6^Department of Phytochemistry and Plant Systematics, Pharmaceutical Division, National Research Centre, Cairo 12622, Egypt. Affiliation ID: 60014618.

***Corresponding authors.**

Mona A. Mohammed^1#*:^ [monaarafamohammed@yahoo.com](mailto:monaarafamohammed@yahoo.com)

[on.ibrahim@nrc.sci.eg](mailto:on.ibrahim@nrc.sci.eg)

Bassant M.M.Ibrahim^2#*^ :bm.ibrahim@nrc.sci.eg,

bmmih1974@gmail.com

Souad E. El-Gengaihi* : [souadgengaihi@hotmail.co.uk](mailto:souadgengaihi@hotmail.co.uk)

**# These authors contributed equally to this work.**


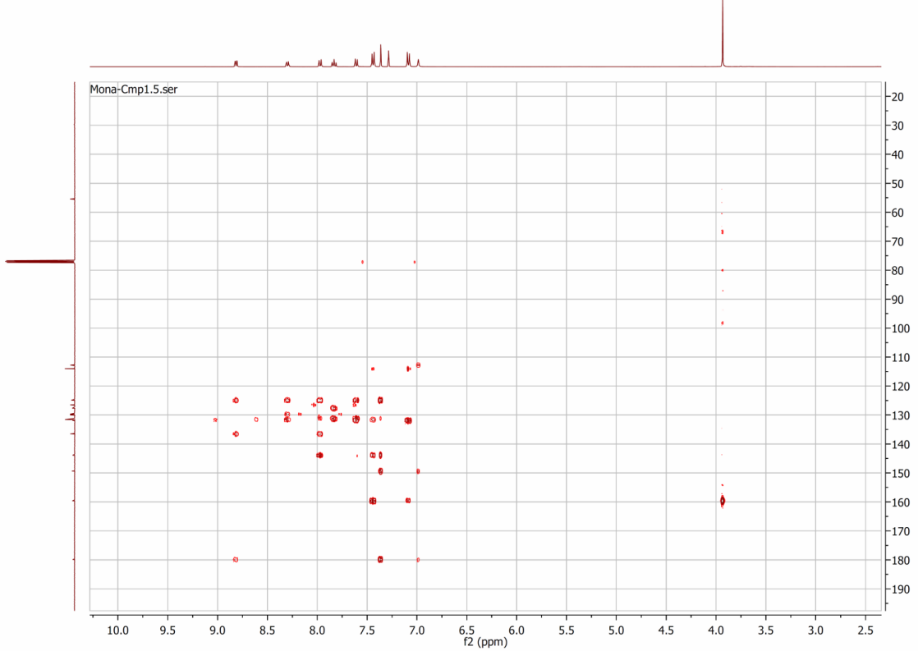

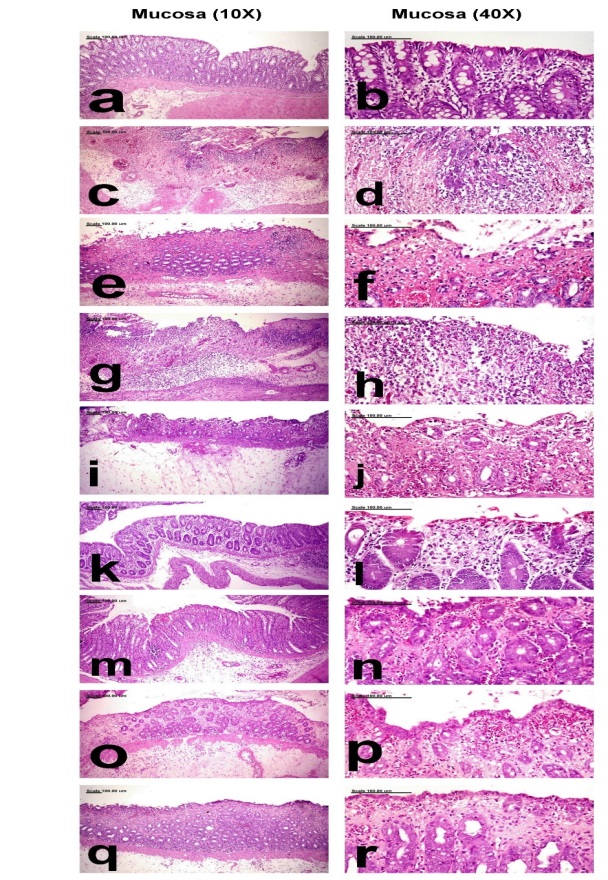

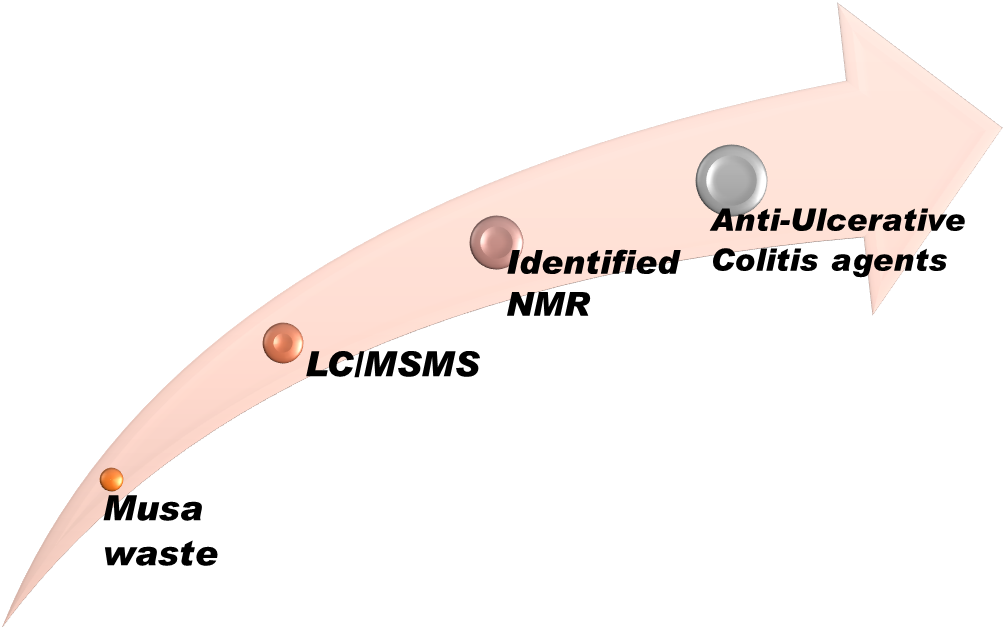


**A potential source of bioactive metabolites as promising anti-UC supplements**

Product


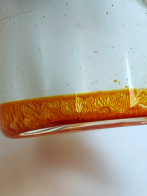

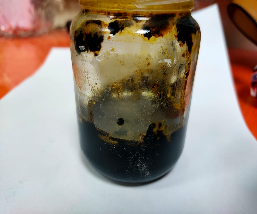


Phenylphenalenones

**Banana peels**

**Leaves & pseudostem**


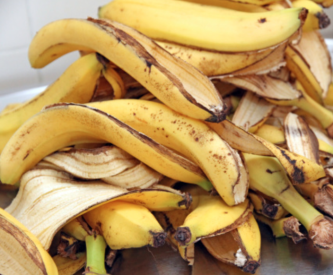


**Fig. S1. Flowchart of a potential source of bioactive metabolites from waste as promising anti-UC supplements new drugs.**

*Photography of banana peels taken by Samsung ES 95 digital camera ,

*Phenylphenalenones diagram done by UPLC (Acquity system, Waters, Milford, USA) coupled to Q-Exactive hybrid MS/MS quadrupole - Orbitrap mass spectrometer (Thermo, Bremen, Germany) Xcalibur version 4.0 software url:( http://tools. thermofisher.com)

*Histopathology images were acquired with a Leica ICC50 HD digital camera attached to a Leica motorized light microscope system url:(

[https://www.leica-microsystems.com)](https://www.leica-microsystems.com)

The whole flowchart was designed using Microsoft Office Powerpoint 2016 url:( http://www.microsoft.com)

Compound 1

Cinnamic acid


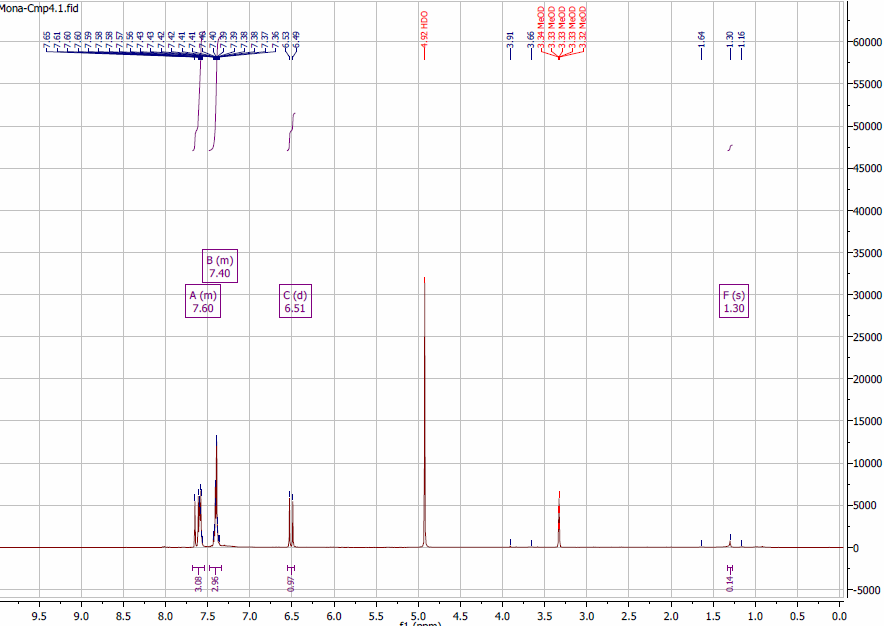

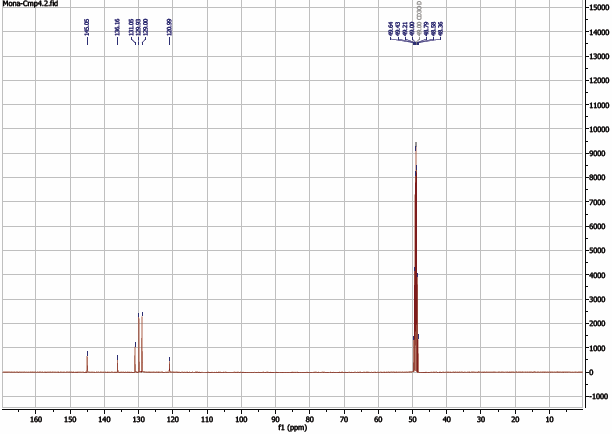


**B**

**A**


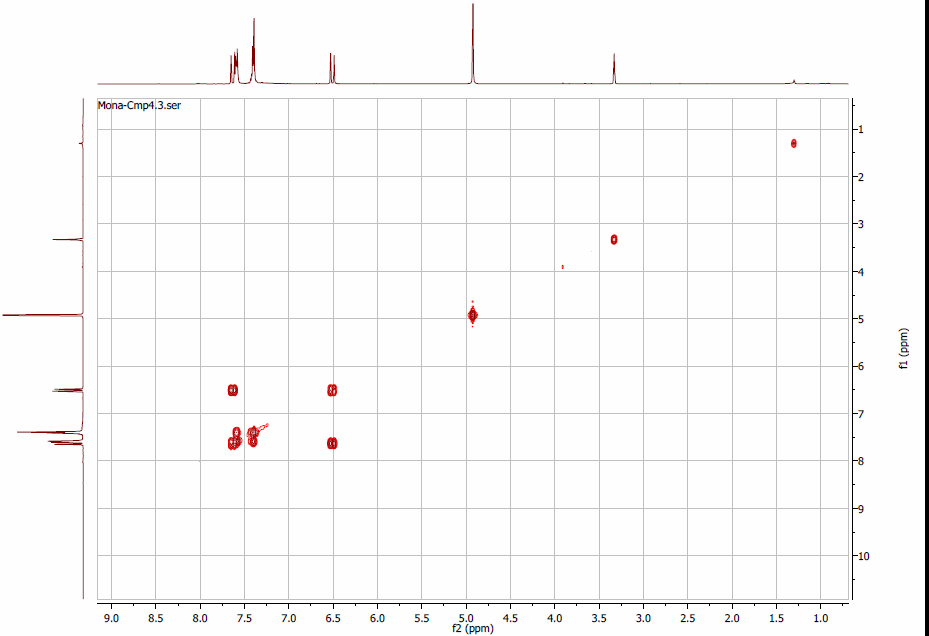

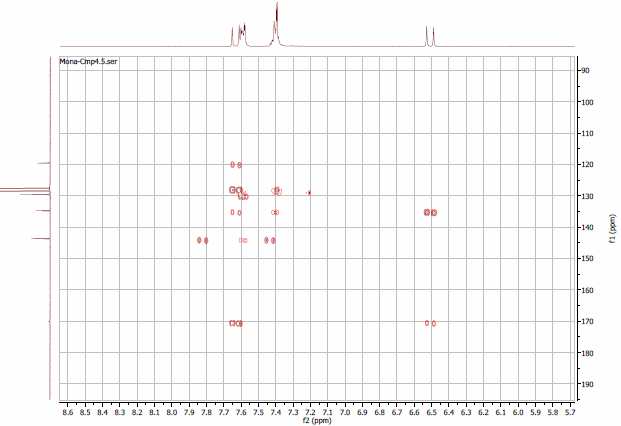

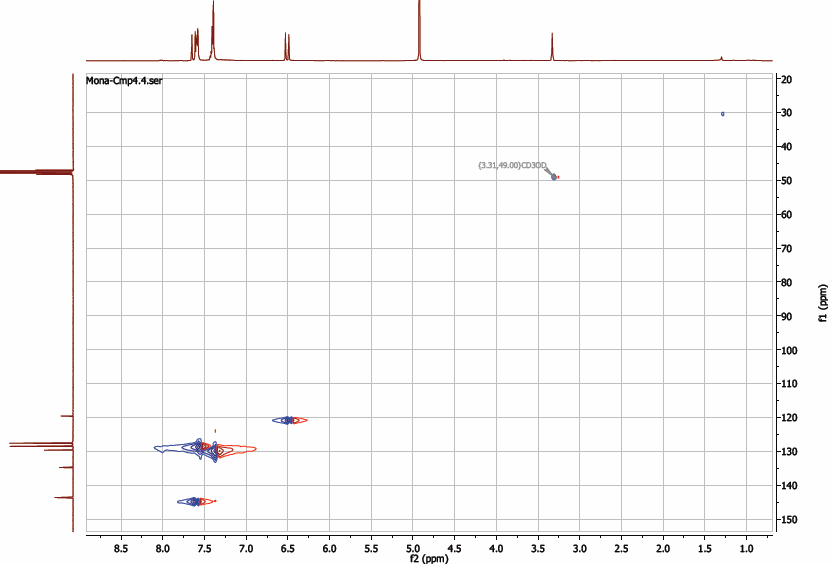


**E**

**D**

**C**

**Fig S2. (A) ^1^H , (B)^13^C, (C)COSY& (D,E)HMBC NMR of Cinnamic acid.**

**Compound 2 Gallic acid**

**B**

**A**


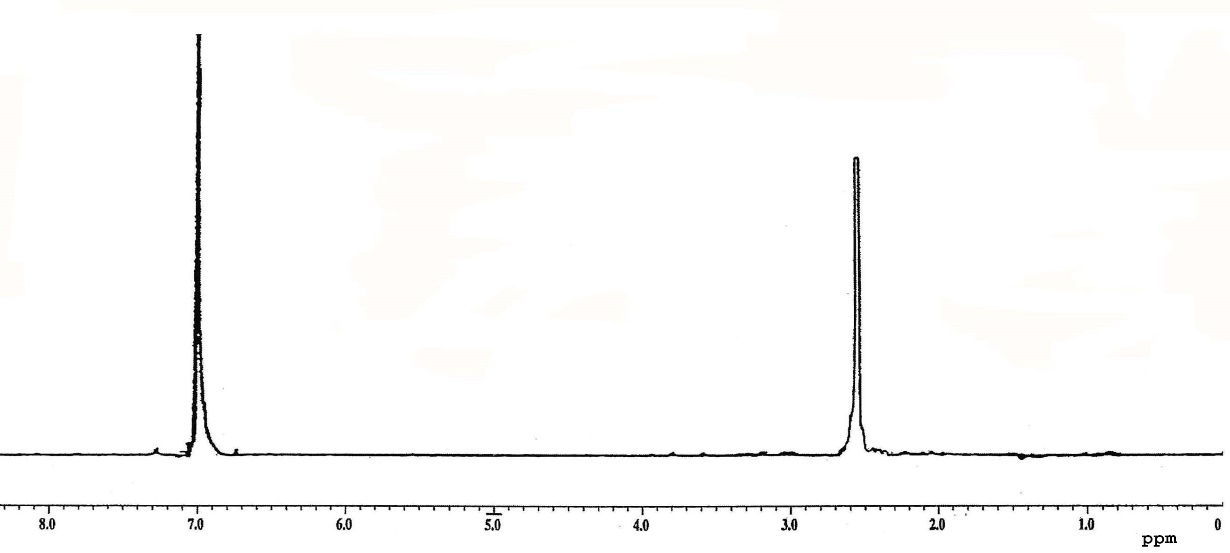

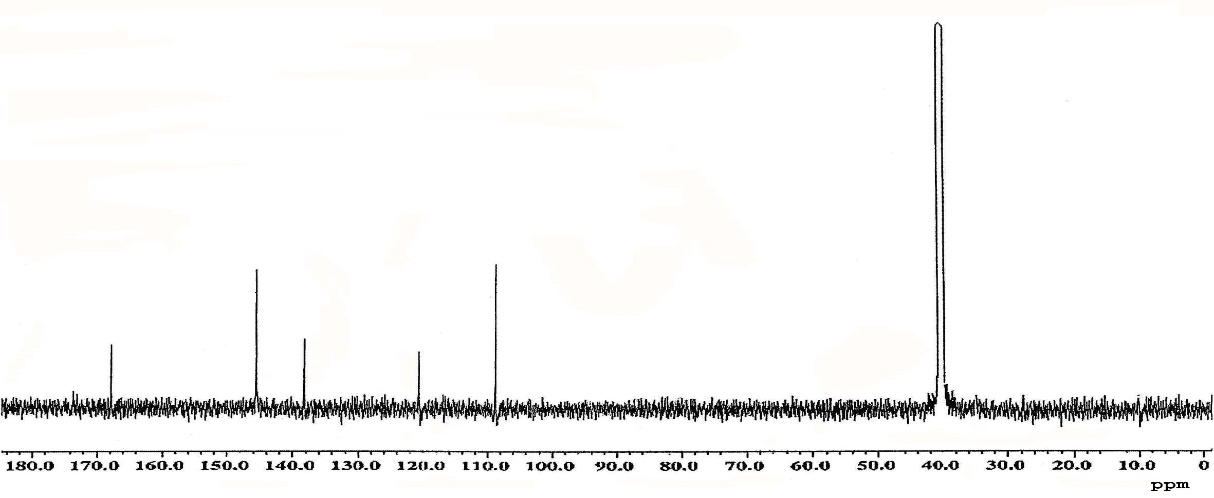


**Fig S3. (A) ^1^H &(B) ^13^C NMR of Gallic acid.**

**Compound 3**

**Quercetin**


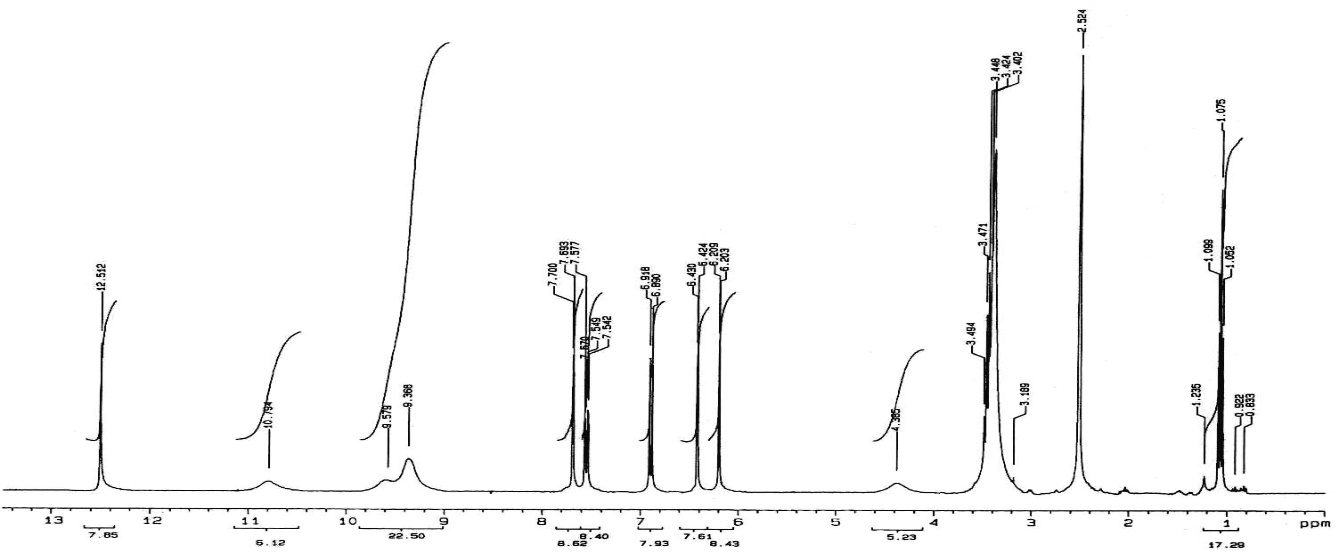


**B**

**A**


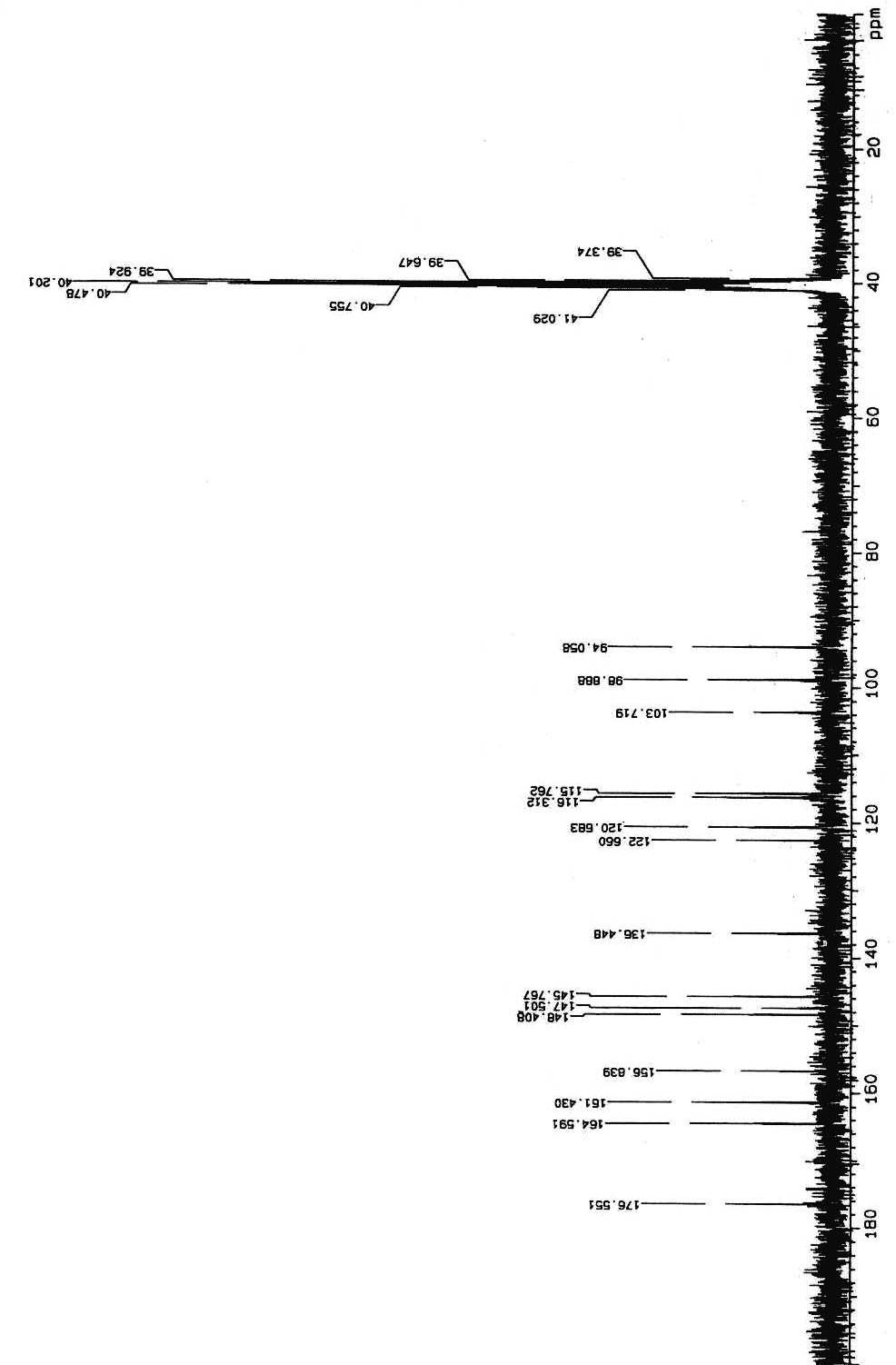


**Fig S4. (A) ^1^H &(B)^13^C NMR of Quercetin.**

**Compound 4**

**Quercetin-3-O-β-D-glucoside**


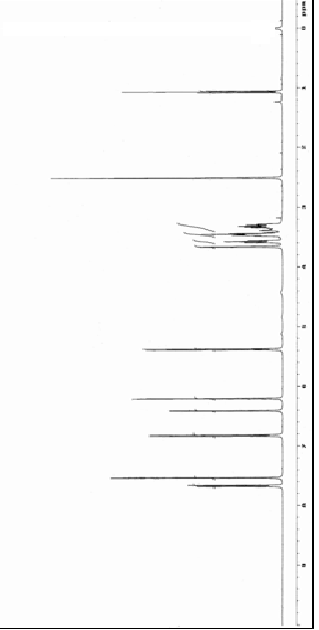


**B**

**A**


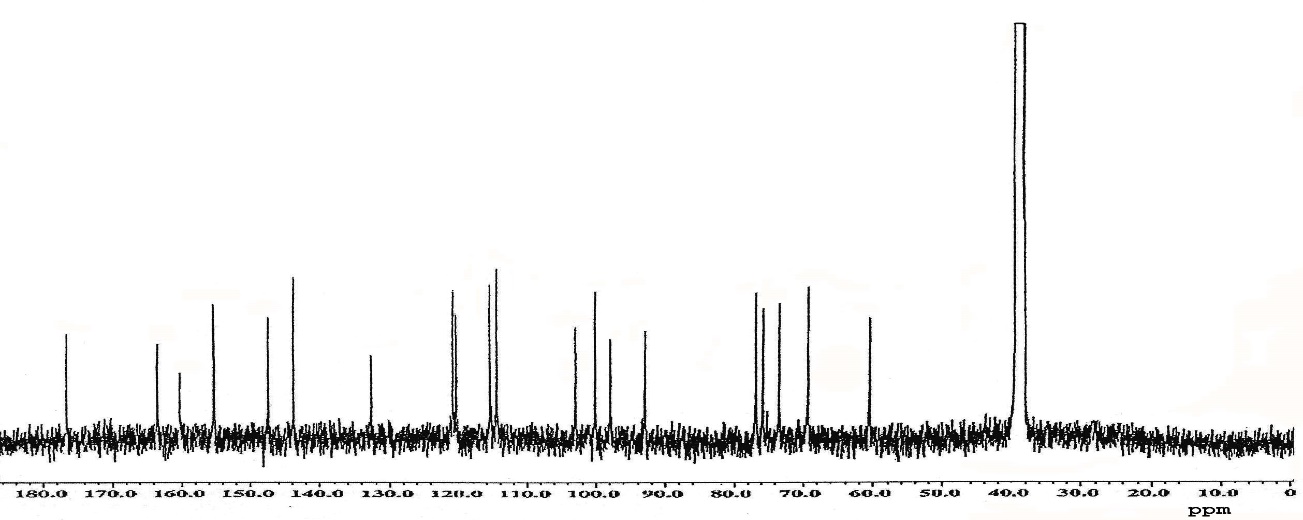


**Fig S5. (A) ^1^H & (B)^13^C NMR of Quercetin- 3-O-β-D-glucoside.**

**Compound 5**

**Luteolin-7-*O*-β-D-glucopyranoside**


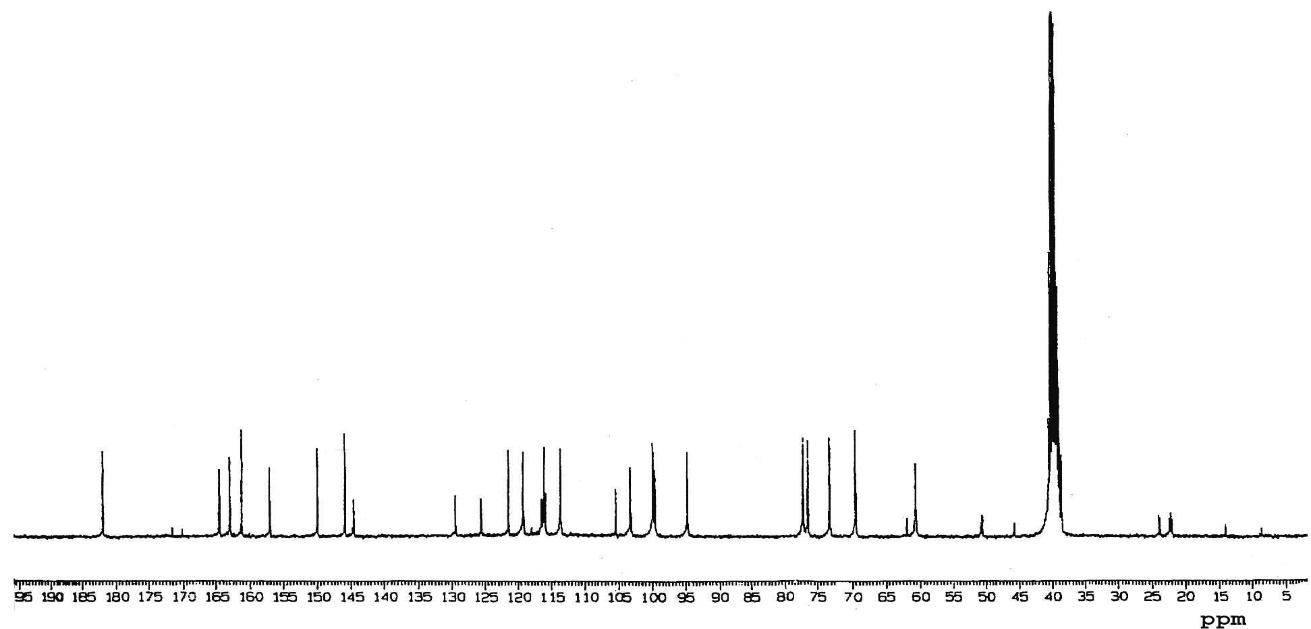

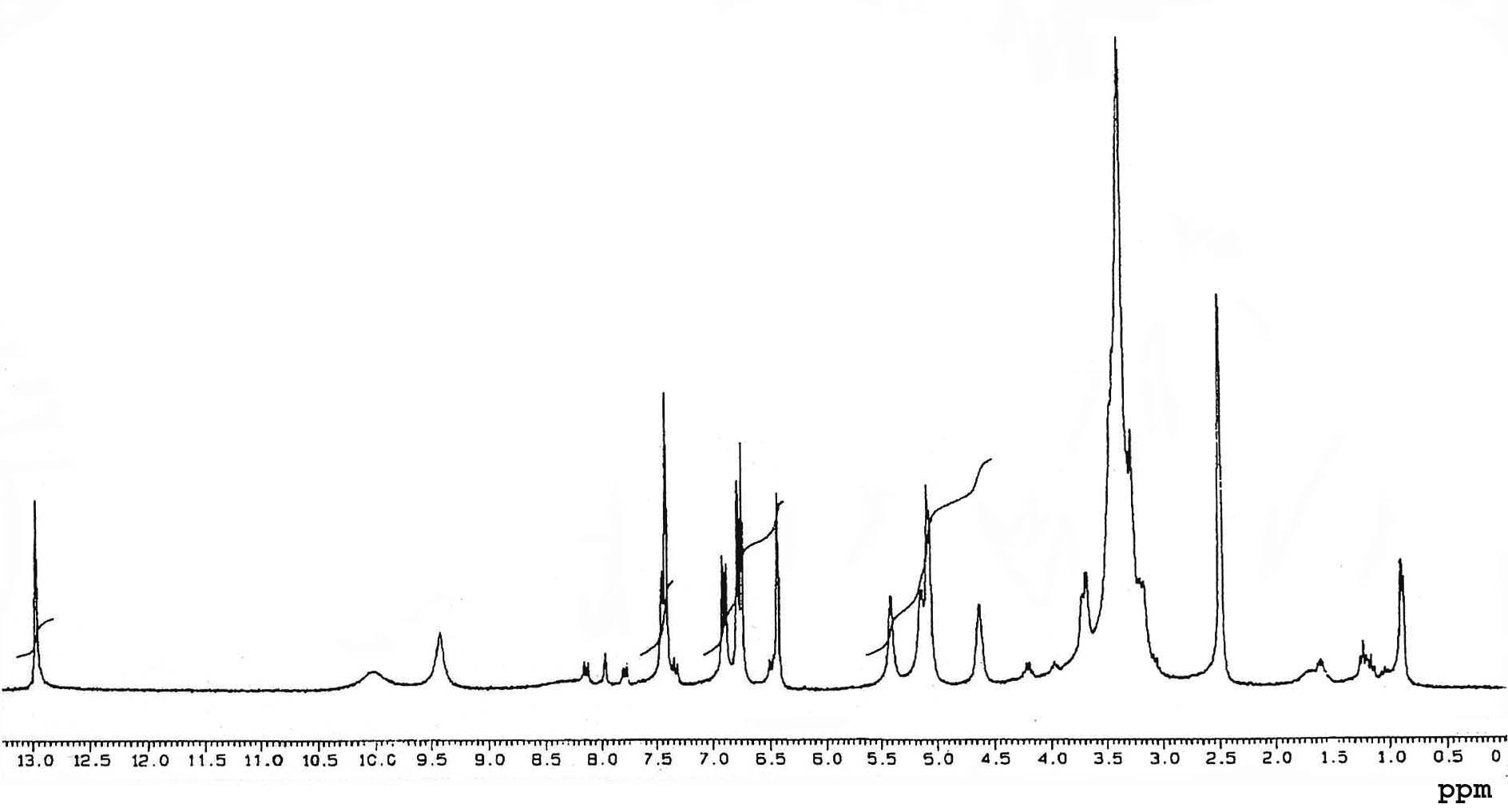


**B**

**A**

**Fig S6. (A) ^1^H & (B)^13^C NMR of Luteolin-7-*O*-β-D-glucopyranoside.**

**Compound 6**

2-hydroxy-4-(4-methoxyphenyl)-1H-phenalen-1-one

**A**

**
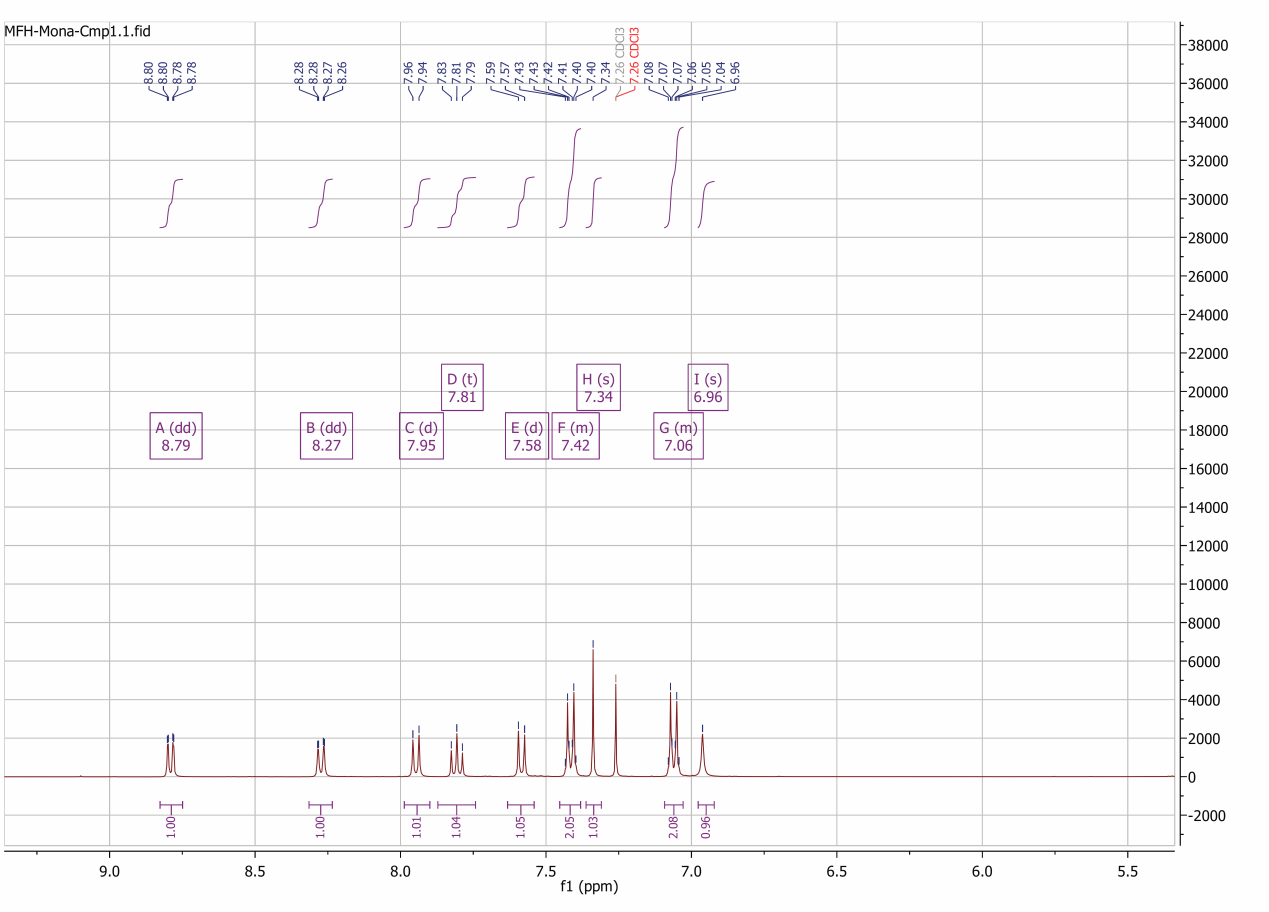
**

**B**

**
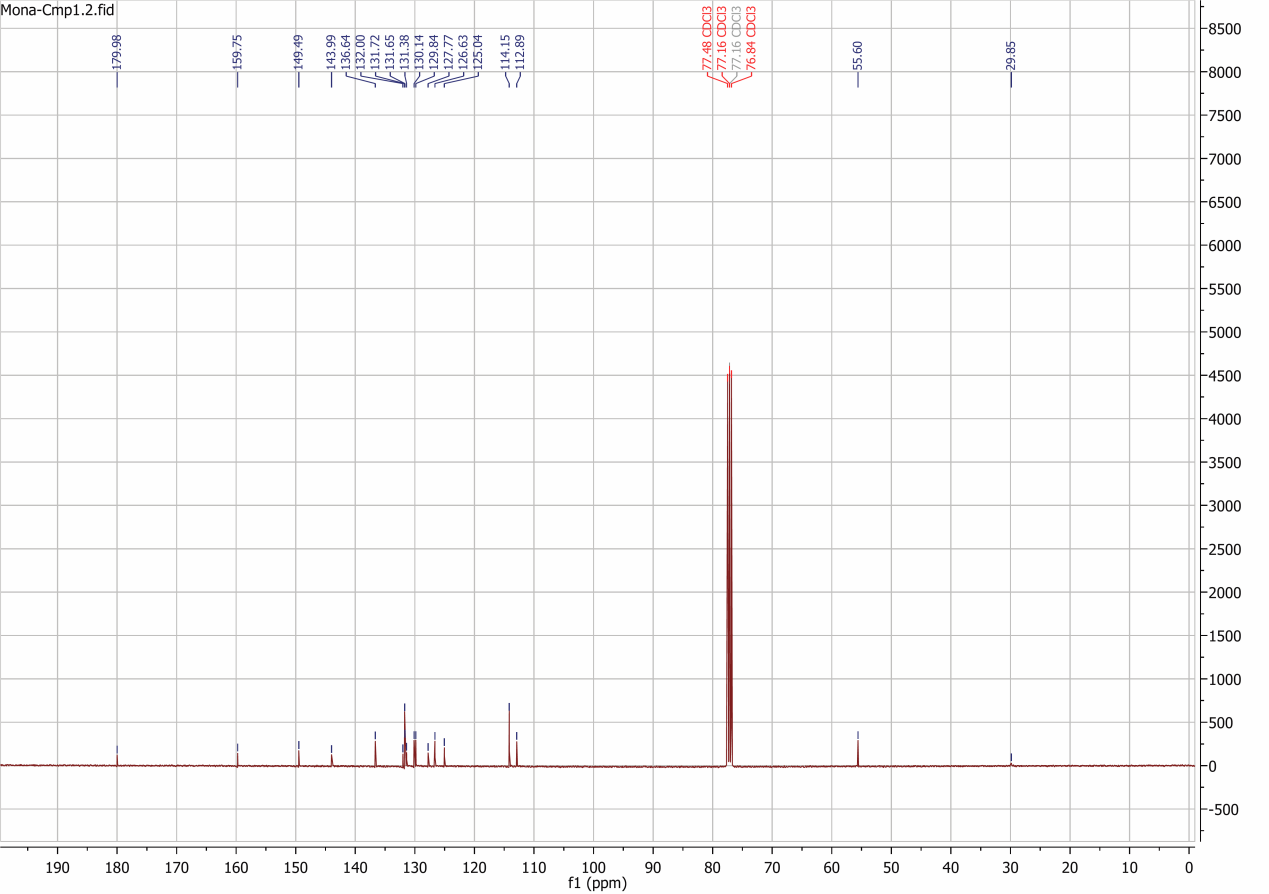
**

**D**

**C**

**
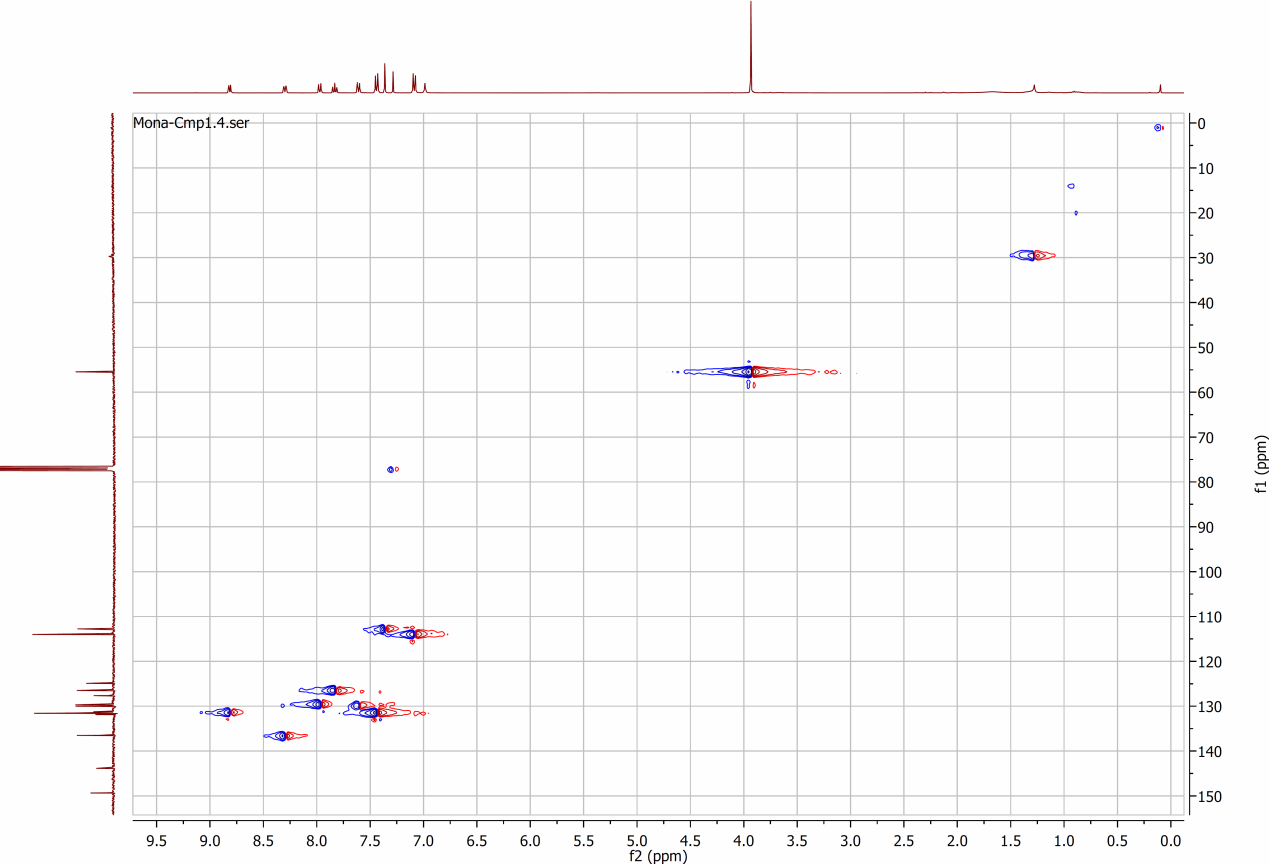
**

**E**

**
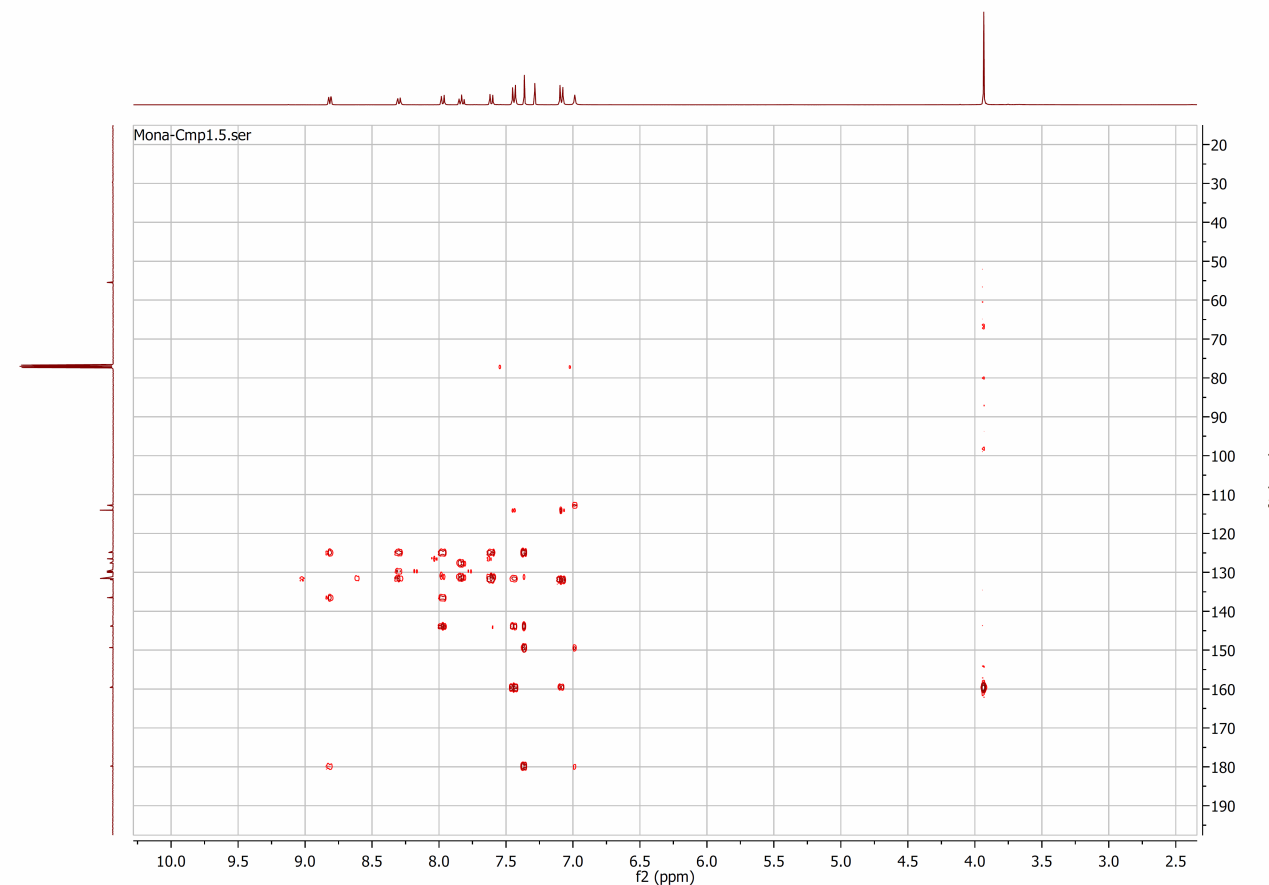
**

**F**

**Fig S7. (A)HRMS (B)^1^H , (C)^13^C, (D)COSY& and (E&F)HMBC NMR of 2-hydroxy-4-(4-methoxyphenyl)-1H-phenalen-1-one.**

**Table S1. Quantitative analysis of phenolic compounds (as** µg/g)**) of different part *Musa acuminata***.

| **phenolic compounds** | Standard (µg/ml) | banana fruit (µg/g) | banana leaves (µg/g) | banana stem µg/g |
| --- | --- | --- | --- | --- |
| **Gallic Acid** | 74.77 | 20.01 | 10.01 | 0.00 |
| **Catechin** | 124.61 | 0.00 | 908.23 | 0.00 |
| **Coffeic Acid** | 62.31 | 106.27 | 0.00 | 25.46 |
| **Syringic Acid** | 49.84 | 0.00 | 0.00 | 0.00 |
| **Rutin** | 149.22 | 50.08 | 3806.15 | 480.14 |
| **Coumaric Acid** | 24.92 | 17.16 | 0.00 | 162.91 |
| **Vanillin** | 62.31 | 0.00 | 878.06 | 23.17 |
| **Ferulic Acid** | 24.92 | 59.60 | 29.76 | 0.00 |
| **Naringin** | 24.92 | 0.00 | 470.93 | 19.16 |
| **Querectin** | 93.46 | 183.59 | 1222.64 | 379.66 |
| **Cinnamic Acid** | 12.46 | 83.63 | 45.91 | 61.32 |
| **Propyl Gallate** | 24.42 | 20.50 | 4713.85 | 34.70 |
| **Luteolin-7-O-β-D-glucopyranoside** | 20.12 | 120.56 | 210.21 | 50.23 |
| **4.7-DihydroxyisoFlavone** | 20.69 | 0.00 | 0.00 | 14.22 |

**Table. S2. *In vitro* IC50 DPPH and ABTS antioxidant activity of different plant parts of Musa acuminata. extracts.**

| **Extracts**  **Concentration**  **ug/ml** | **DPPH IC_50_** | **ABTS IC_50_** |
| --- | --- | --- |
| **Pet-leaves** | 332.0 ± 0.04 | 144.30 ± 0.85 |
| **CHCL3- leaves** | 3.65 ± 0.25 | 29.64 ±1.02 |
| **EtOAc-Leaves** | 7.73 ± 0.45 | 25.90 ±2.36 |
| **BuOH-Leaves** | **5.85** ± 0.74 | **14.92** ±0.55 |
| **Pet-stem** | 393.5 ± 0.58 | 331.4 ± 0.86 |
| **CHCL3- stem** | 78.59 ± 026 | 243.20 ± 0.25 |
| **EtOAc-stem** | 7.17 ± 0.15 | 54.02 ± 0.25 |
| **BuOH-stem** | **13.17** ± 0.25 | **41.48** ± 0.45 |
| **Pet-fruit** | 71.87 ± 0.36 | 88.84 ±0.15 |
| **CHCL3- fruit** | 84.89 ± 0.85 | 452.80 ± 0.48 |
| **EtOAc-fruit** | 5.91 ± 012 | 78.97 ±0.48 |
| **BuOH-fruit** | **9.99** ± 0.32 | **12.08** ±0.98 |
| **vit C** | 2.62 ± 0.25 | 0.29 ± 0.58 |
| **trolox** | 3.47 ± 0.78 | 1.01 ± 0.26 |

**Table S3.The main constituents of the essential oil peel waste fruit *Musa acuminata***.

| **No** | **Name compounds** | **Formula** | **Rt** | **Area Sum %** |  |
| --- | --- | --- | --- | --- | --- |
| 1 | 3-methyl butanol | C_5_H_12_O | 3.15 | 0.89 | |
| 2 | Isobutyl acetate | C_6_H_12_O_2_ | 3.81 | 0.34 | |
| 3 | 2-Pentanol, acetate | C_7_H_14_O_2_ | 5.51 | 1.02 | |
| 4 | 1-Butanol, 3-methyl-, acetate | C_7_H_14_O_2_ | 6.24 | 2.99 | |
| 5 | Butanoic acid, 2-methylpropyl ester | C_8_H_16_O_2_ | 8.70 | 2.46 | |
| 6 | Butanoic acid, butyl ester | C_8_H_16_O_2_ | 10.08 | 1.42 | |
| 7 | Isobutyl isovalerate | C_9_H_18_O_2_ | 10.43 | 0.51 | |
| 8 | Butanoic acid, 1-methylbutyl ester | C_9_H_18_O_2_ | 11.11 | 3.77 | |
| 9 | 2-Heptanol, acetate | C_9_H_18_O_2_ | 11.74 | 2.55 | |
| 10 | Butanoic acid, 3-methyl-, butyl ester | C_9_H_18_O_2_ | 11.86 | 0.50 | |
| **11** | **isoamyl isobutyrate** | **C_9_H_18_O_2_** | **12.25** | **18.3** | |
| **12** | **Isovaleric acid** | **C_10_H_20_O_2_** | **13.99** | **8.06** | |
| 13 | 1,3,8-p-Menthatriene | C_10_H_14_ | 14.13 | 0.49 | |
| **14** | Butanoic acid, hexyl ester | C_10_H_20_O_2_ | 17.07 | 1.09 | |
| 15 | Butanoic acid, 1-cyclopentylethyl ester | C_11_H_20_O_2_ | 17.87 | 3.56 | |
| 16 | Butanoic acid, 3-methyl-, hexyl ester | C_11_H_22_O_2_ | 18.78 | 1.22 | |
| 17 | Isopentyl hexanoate | C_11_H_22_O_2_ | 19.06 | 0.75 | |
| 18 | Butanoic acid, 1-ethenylhexyl ester | C_12_H_22_O_2_ | 23.26 | 0.48 | |
| 19 | Hexanoic acid, undec-10-enyl ester | C_17_H_32_O_2_ | 23.59 | 0.25 | |
| 20 | Z,Z,Z-4,6,9-Nonadecatriene | C_19_H_34_ | 24.60 | 0.39 | |
| 21 | Cyclohexane, ethylidene- | C_8_H_14_ | 24.81 | 0.93 | |
| 22 | (Z)-5-Octen-1-ol | C_8_H_16_O | 25.15 | 0.35 | |
| 23 | cis-.beta.-Farnesene | C_15_H_24_ | 25.77 | 0.24 | |
| 24 | β-Cubebene | C_15_H_24_ | 26.49 | 0.45 | |
| 25 | **Myristicine** | **C_11_H_12_O_3_** | **27.77** | **9.31** | |
| 26 | Benzene, 1,2,3-trimethoxy-5-(2-propenyl)- | C_12_H_16_O_3_ | 28.81 | 1.39 | |
| **27** | Bicyclo[8.2.0]dodecane, 11,11-dimethyl- | C_14_H_26_ | 29.03 | 0.23 | |
| 28 | Cyclohexane, 1-methylene-3-(1-methylethenyl)-, (R)- | C_10_H_16_ | 30.86 | 0.4 | |
| 29 | Apiol | C_12_H_14_O_4_ | 31.11 | 0.29 | |
| 30 | 1,1'-Biphenyl, 3,4-diethyl- | C_16_H_18_ | 33.30 | 0.33 | |
| 31 | Phthalic acid, butyl undecyl ester | C_23_H_36_O_4_ | 41.30 | 0.85 | |
| 32 | Palmitoleic acid | C_16_H_30_O_2_ | 43.86 | 0.6 | |
| 33 | **n-Hexadecanoic acid** | **C_16_H_32_O_2_** | **44.76** | **22.65** | |
| 34 | 11-Octadecenoic acid, methyl ester | C_19_H_36_O_2_ | 48.09 | 0.56 | |
| 35 | Phytol | C_20_H_40_O | 48.60 | 1.65 | |
| **36** | 9,12-Octadecadienoic acid (Z,Z)- | C_18_H_32_O_2_ | 49.04 | 4.21 | |
| 37 | 9,12,15-Octadecatrienoic acid, (Z,Z,Z)- | C_18_H_30_O_2_ | 49.20 | 4.57 | |

**Table S4. Body weight changes during the Acute toxicity study of MA leaves , pseudostem and fruit peels extracts.**

| **Group** | | **Negative control** | **MA leaves**  **(5000mg/kg)** | **MA pseudostem**  **(5000mg/kg)** | **MA fruit peels (5000mg/kg)** |
| --- | --- | --- | --- | --- | --- |
| Onset of Body weight measurement | Baseline body  weight (gm) | 160.6 ± 1.07 | 160.66 ± 1.07 | 160.66 ± 1.07 | 160.66 ± 1.07 |
|  | Body weight (gm)  After two weeks | 172.36 ± 5.8 | 1706 ± 3.7 | 177.86 ± 4.6 | 1666 ± 6.3 |
| % of body weight increase  after 2 weeks | | 7.286 ± 0.16 | 5.856 ± 0.43* | 3.366 ± 0.2^@*#^ | 10.76 ± 0.57^@^ |

Results are expressed as means of body weights of rats (gm)+ SE after fourteen days of single oral dose of MA. n = 5; Data were analysed using one way analysis of variance (ANOVA) followed by Tukey Kramer’s multiple. comparison test; Significance was considered at P ≤ 0.05.

@Significantly different from negative control group, *Significantly different from MA fruit peels group,# Significantly different from MA leaves group

**Table S5.Body weight changes during the Sub chronic toxicity and Efficacy study of MA leaves ,pseudostem and fruit peels extracts**

| **Onset**  **Groups** | **Baseline body weight (gm)** | **Body weight (gm)After Two Successive weeks** |
| --- | --- | --- |
| **Negative control** | 155.8+2.8 | 163.3+3.33 |
| **MA leaves (250 mg/kg)** |  | 169+5.5 |
| **MA leaves(500 mg/kg)** |  | 166.3+4.48 |
| **MA pseudostem(250 mg/kg)** |  | 156.7+4.41 |
| **MA pseudostem(500 mg/kg)** |  | 150.7+3.84 |
| **MA fruit peels(250 mg/kg)** |  | 162.7+1.3 |
| **MA fruit peels(500 mg/kg)** |  | 167.7+1.45 |

Results are expressed as means of body weights of rats in gm + SE. n = 8; Data were analysed using one way analysis of variance (ANOVA) followed by Tukey Kramer’s multiple comparison test. No significant difference detected among groups.

**Raw Data**

**Raw data of the Phytochemical study**


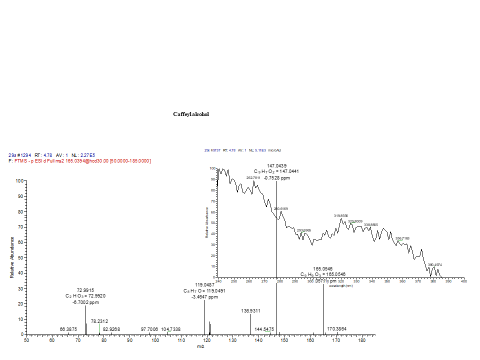


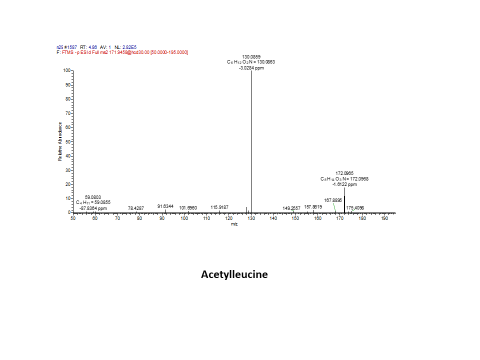

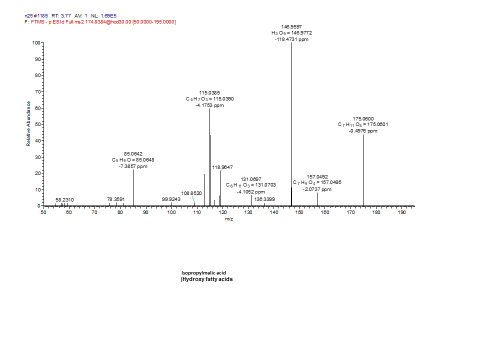

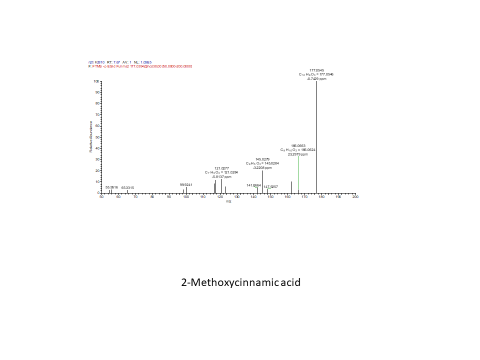

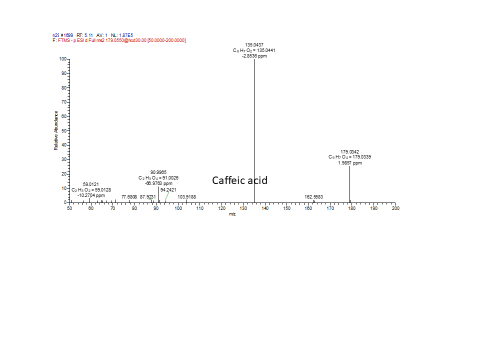

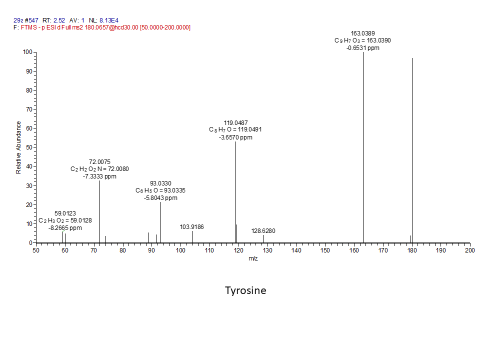

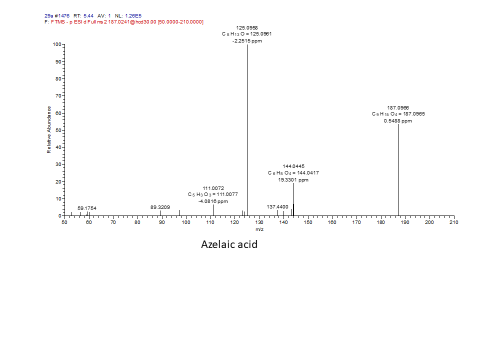

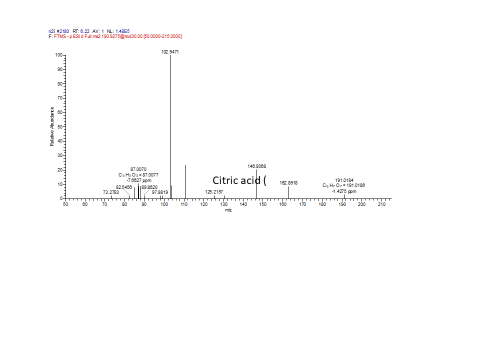

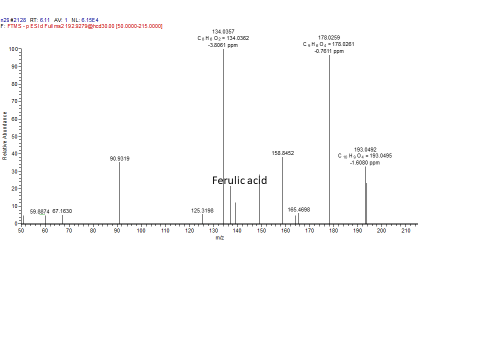

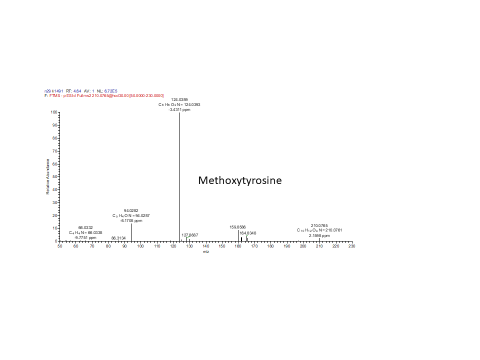

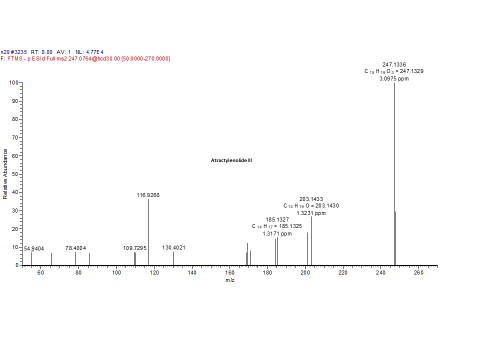

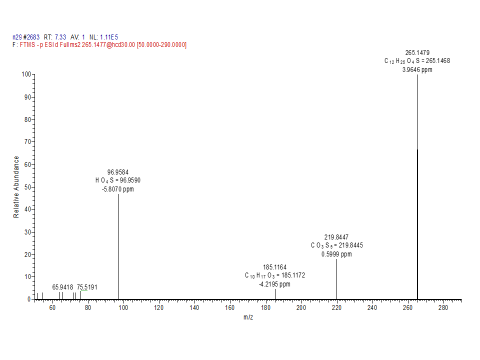

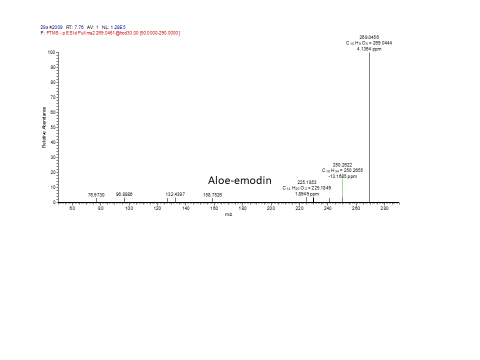

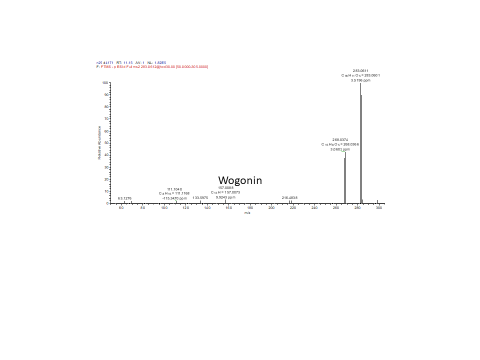

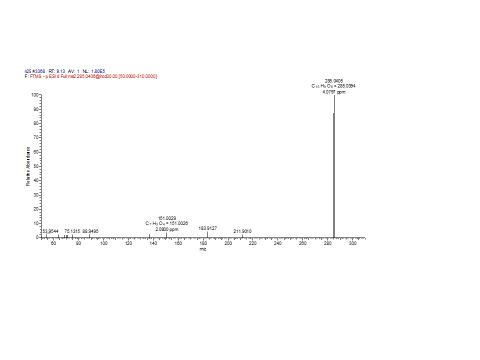

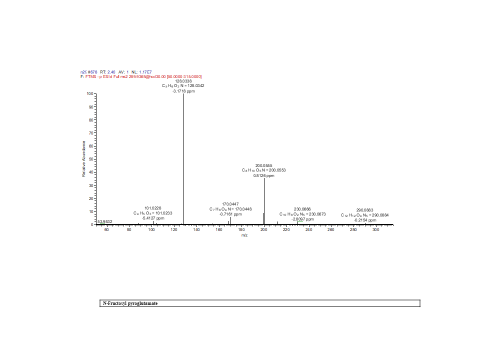

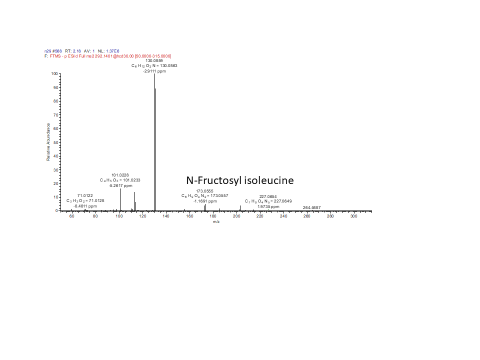

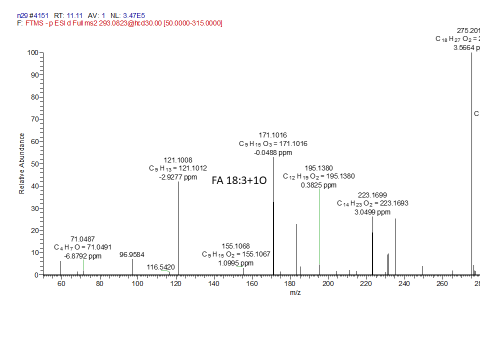

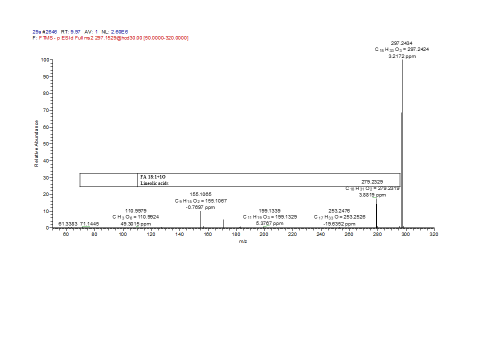

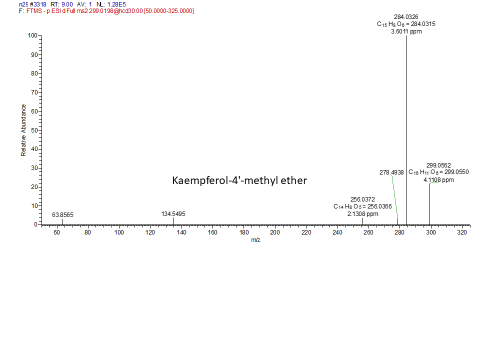

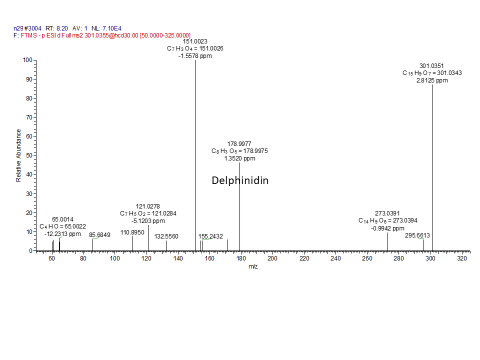

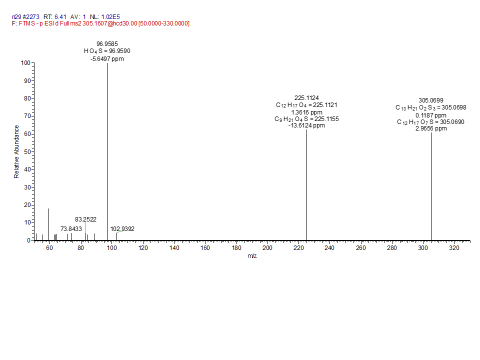

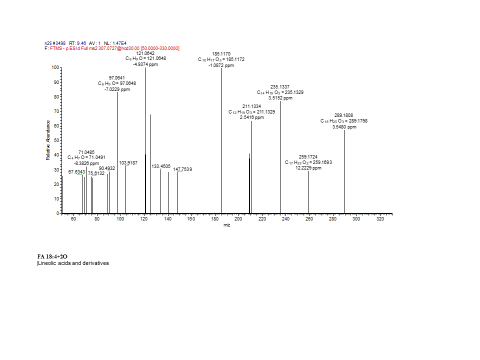

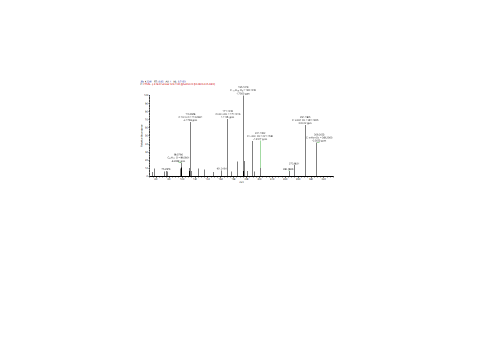

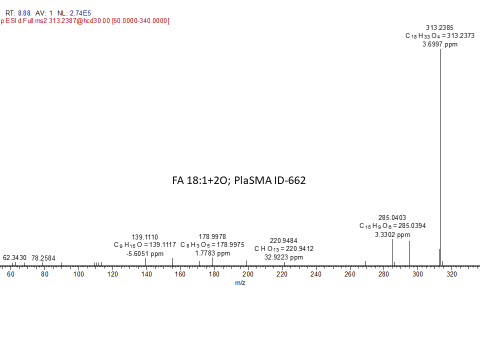

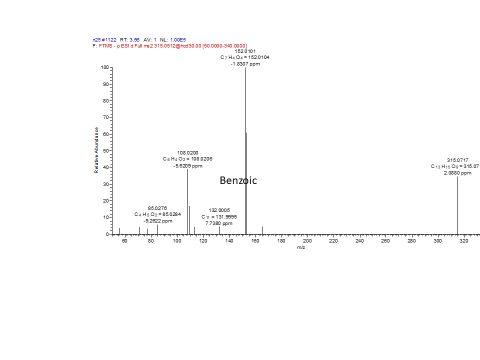

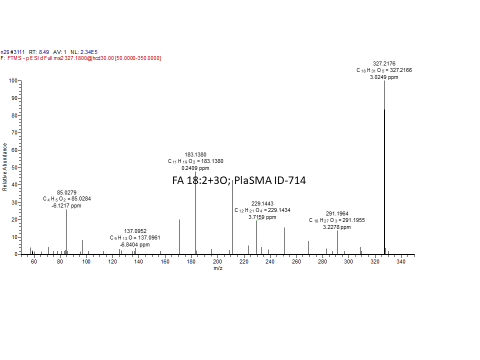

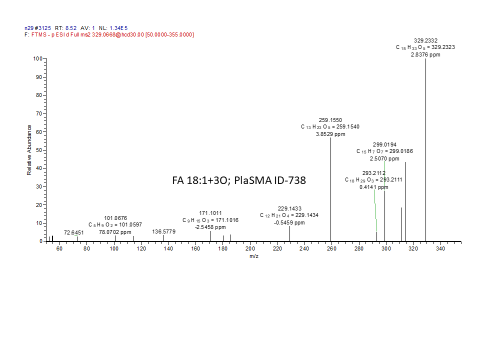

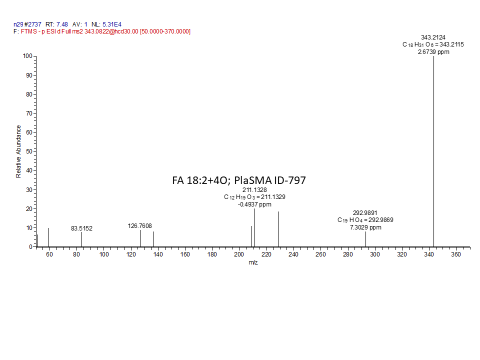

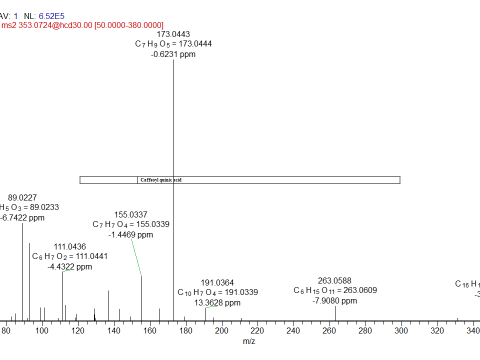

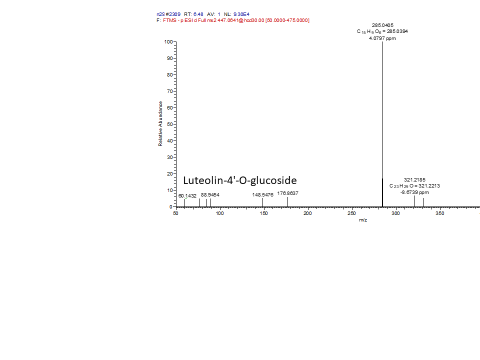

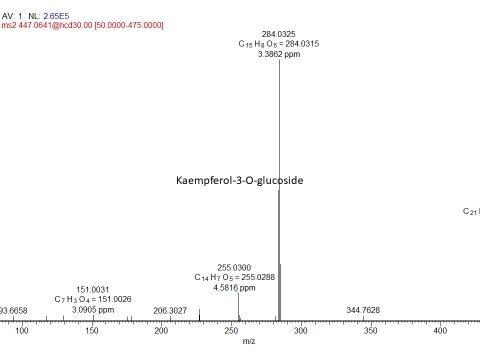

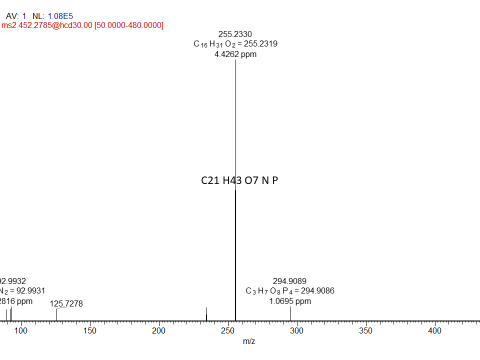

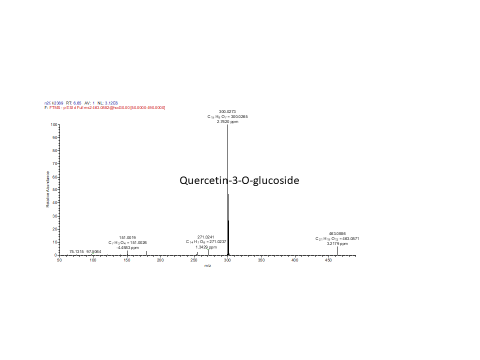

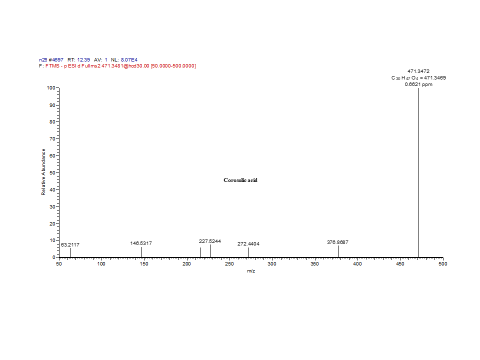

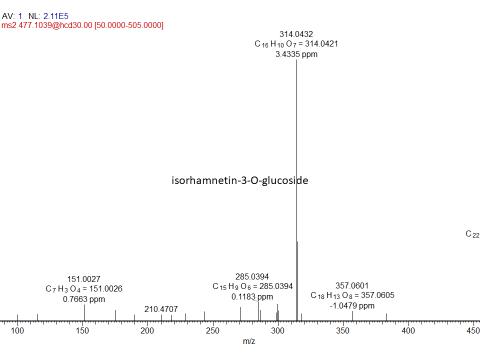

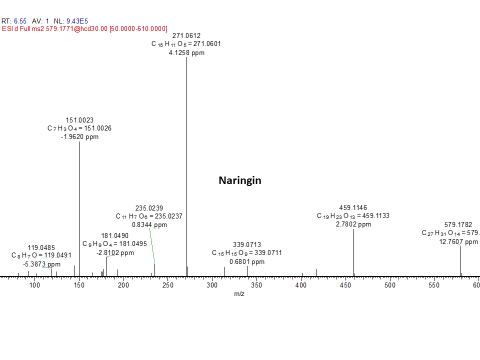

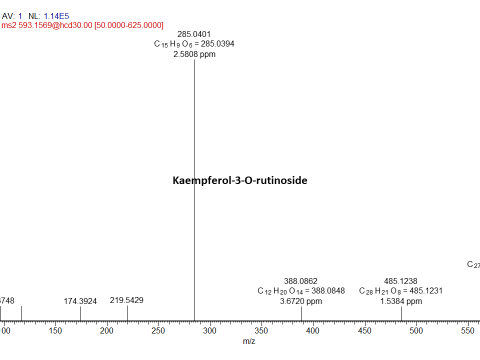

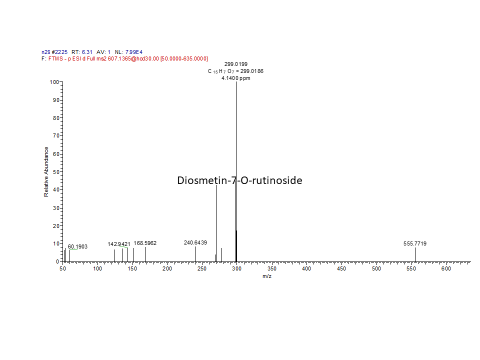

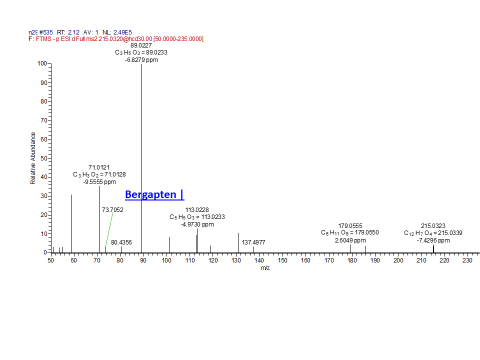

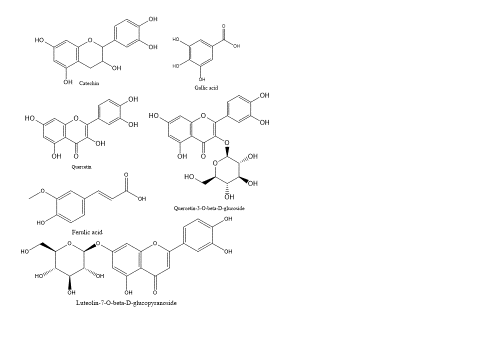

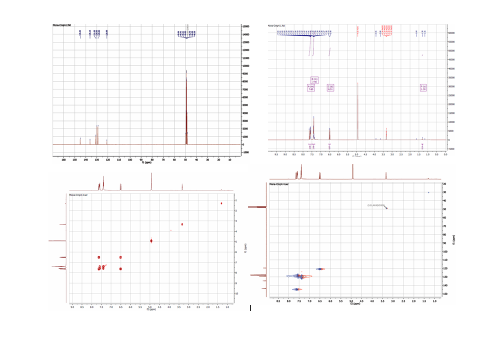

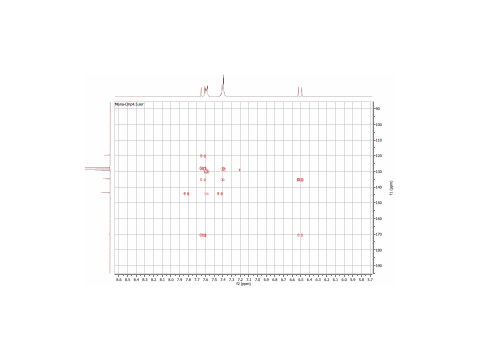

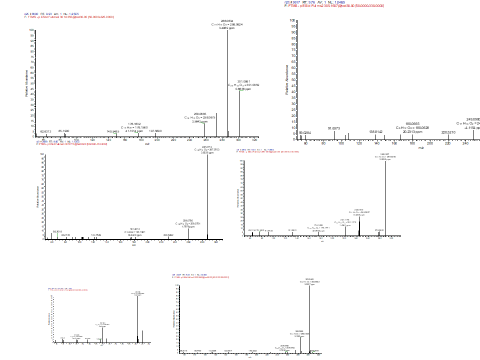


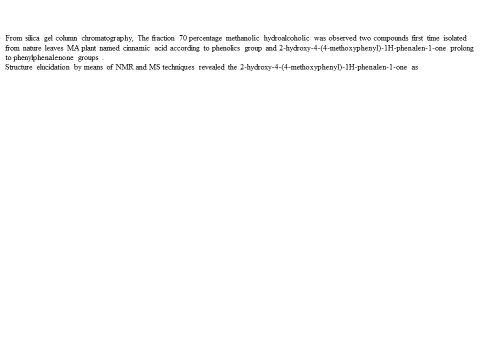


**Efficacy study raw data**

**Macroscopic Ulcer grading raw data**

|  | Number | Severity | Index | % Protection | |
| --- | --- | --- | --- | --- | --- |
| Positve control | 5 | 5 | 1100 |  |  |
|  | 5 | 5 | 1100 |  |  |
|  | 5 | 5 | 1100 |  |  |
|  | 5 | 5 | 1100 |  |  |
|  | 5 | 5 | 1100 |  |  |
|  | 5 | 5 | 1100 |  |  |
|  | 5 | 5 | 1100 |  |  |
|  | 5 | 5 | 1100 |  |  |
| Mean | 5 | 5 | 1100 |  |  |
| Prednisolone (5mg/kg) | 1 | 0 | 635 | 42.27273 |  |
|  | 0 | 0 | 625 | 43.18182 |  |
|  | 0 | 0 | 625 | 43.18182 |  |
|  | 2 | 1 | 655 | 40.45455 |  |
|  | 1 | 0 | 635 | 42.27273 |  |
|  | 2 | 1 | 655 | 40.45455 |  |
|  | 1 | 1 | 645 | 41.36364 |  |
|  | 1 | 1 | 645 | 41.36364 |  |
| Mean | 1 | 0.5 | 640 | 41.81818 |  |
| Leaves (250 mg/kg) | 4 | 2 | 935 | 15 |  |
|  | 5 | 5 | 975 | 11.36364 |  |
|  | 3 | 3 | 935 | 15 |  |
|  | 4 | 2 | 935 | 15 |  |
|  | 0 | 0 | 875 | 20.45455 |  |
|  | 2 | 2 | 915 | 16.81818 |  |
|  | 5 | 4 | 965 | 12.27273 |  |
|  | 3 | 3 | 935 | 15 |  |
| Mean | 3.25 | 2.625 | 933.75 | 15.11364 |  |
| Leaves (500mg/kg) | 3 | 2 | 800 | 27.27273 |  |
|  | 3 | 2 | 800 | 27.27273 |  |
|  | 2 | 2 | 790 | 28.18182 |  |
|  | 0 | 0 | 750 | 31.81818 |  |
|  | 2 | 2 | 790 | 28.18182 |  |
|  | 4 | 3 | 820 | 25.45455 |  |
|  | 2 | 0 | 770 | 30 |  |
|  | 0 | 0 | 750 | 31.81818 |  |
| Mean | 2 | 1.375 | 788.5714 | 28.31169 |  |
| Pseudo Stem (250 mg/kg) | 3 | 2 | 800 | 27.27273 |  |
|  | 2 | 2 | 790 | 28.18182 |  |
|  | 3 | 2 | 800 | 27.27273 |  |
|  | 0 | 0 | 750 | 31.81818 |  |
|  | 1 | 2 | 780 | 29.09091 |  |
|  | 0 | 0 | 750 | 31.81818 |  |
|  | 4 | 2 | 810 | 26.36364 |  |
|  | 3 | 3 | 810 | 26.36364 |  |
| Mean | 2 | 1.625 | 786.25 | 28.52273 |  |
| PseudoStem (500 mg/kg) | 2 | 2 | 665 | 39.54545 |  |
|  | 2 | 1 | 655 | 40.45455 |  |
|  | 0 | 0 | 625 | 43.18182 |  |
|  | 0 | 0 | 625 | 43.18182 |  |
|  | 3 | 3 | 685 | 37.72727 |  |
|  | 4 | 2 | 685 | 37.72727 |  |
|  | 0 | 0 | 625 | 43.18182 |  |
|  | 4 | 2 | 685 | 37.72727 |  |
| Mean | 1.875 | 1.25 | 656.25 | 40.34091 |  |
| Fruit peels (250 mg/kg) | 1 | 1 | 645 | 41.36364 |  |
|  | 2 | 1 | 655 | 40.45455 |  |
|  | 3 | 2 | 675 | 38.63636 |  |
|  | 0 | 0 | 625 | 43.18182 |  |
|  | 0 | 0 | 625 | 43.18182 |  |
|  | 0 | 0 | 625 | 43.18182 |  |
|  | 2 | 1 | 655 | 40.45455 |  |
|  | 3 | 2 | 675 | 38.63636 |  |
| Mean | 1.375 | 0.875 | 647.5 | 41.13636 |  |
| Fruit peels(500 mg/kg) | 1 | 1 | 520 | 52.72727 |  |
|  | 1 | 0 | 510 | 53.63636 |  |
|  | 0 | 0 | 500 | 54.54545 |  |
|  | 0 | 0 | 500 | 54.54545 |  |
|  | 0 | 0 | 500 | 54.54545 |  |
|  | 0 | 0 | 500 | 54.54545 |  |
|  | 2 | 1 | 530 | 51.81818 |  |
|  | 1 | 1 | 520 | 52.72727 |  |
|  | 2 | 2 | 540 | 50.90909 |  |
| Mean | 0.777778 | 0.555556 | 513.3333 | 53.33333 |  |

**C-Reactive Protein raw data**

|  | Negative control | Positive control (Acetic acid (2ml)8%) | Prednisolone (5 mg/kg) | leaves (250mg/kg) | leaves (500mg/kg) |
| --- | --- | --- | --- | --- | --- |
|  | 1.34 | 3.36 | 2.21 | 2.56 | 2.68 |
|  | 1.3 | 2.99 | 2.16 | 2.73 | 2.65 |
|  | 1.17 | 2.69 | 2.22 | 2.7 | 2.94 |
|  | 1.56 | 3.45 | 2.15 | 2.59 | 2 |
|  | 1.38 | 2.98 | 2.23 | 2.75 | 2.79 |
|  | 1.25 | 2.98 | 2.19 | 2.54 | 2.86 |
|  | 1.8 | 2.6 | 2.2 | 2.77 | 2.1 |
|  | 1.1 | 3.5 | 2.12 | 2.48 | 2.88 |
| Mean | 1.3625 | 3.06875 | 2.185 | 2.64 | 2.6125 |
| Std. Deviation | 0.2248 | 0.3383 | 0.03817 | 0.1103 | 0.3616 |
| Std. Error of Mean | 0.07948 | 0.1196 | 0.0135 | 0.03901 | 0.1278 |
| Pseudostem  (250mg/kg) | Pseudostem  (500mg/kg) | fruit peels(250 mg/Kg) | | fruit peels(500mg/kg) | |
| 2.75 | 1.97 | 2.79 | | 1.83 | |
| 2.3 | 2.89 | 2.39 | | 2.72 | |
| 1.3 | 1.99 | 2.27 | | 1.9 | |
| 2.7 | 2.59 | 3.21 | | 1.86 | |
| 2.72 | 2.56 | 2.43 | | 1.54 | |
| 2.04 | 1.89 | 2.94 | | 1.1 | |
| 3.61 | 1.9 | 2.47 | | 1.94 | |
| 2.1 | 1.44 | 2.1 | | 2.21 | |
| **Mean** 2.44 | **Mean**  2.15375 | **Mean**  2.575 | | **Mean**  1.8875 | |
| **Std. Deviation 0**.6767 | **Std Ddeviation** 0.4783 | **Std Ddeviation**  0.3718 | | **Std Ddeviation**  0.4698 | |
| **Std. Error of Mean** 0.2393 | **Std. Error of Mean** 0.1691 | **Std. Error of Mean** 0.1314 | | **Std. Error of Mean** 0.1661 | |

**Interleukin β6 Levels raw data**

|  | Negative control | Acetic acid (2ml)8% | Prednisolone (5 mg/kg) | leaves (250 mg/kg) | leaves 500 |
| --- | --- | --- | --- | --- | --- |
|  | 39.8 | 110.8 | 57 | 80.4 | 76.8 |
|  | 39.3 | 103.2 | 53.6 | 99.9 | 70.2 |
|  | 48.1 | 116.4 | 55.2 | 82.9 | 67.3 |
|  | 40.5 | 120.7 | 52.2 | 76.2 | 63.4 |
|  | 45.2 | 110.4 | 55.3 | 53.5 | 56.3 |
|  | 50.4 | 116 | 53.7 | 96.7 | 72.2 |
|  | 50.1 | 109.7 | 55 | 83 | 67 |
|  | 37.2 | 127.2 | 54.3 | 72 | 82.2 |
| **Mean** | 43.825 | 114.3 | 54.5375 | 80.575 | 69.425 |
| **Std. Deviation** | 5.27 | 7.431 | 1.434 | 14.49 | 7.972 |
| **Std. Error of mean** | 1.863 | 2.627 | 0.5071 | 5.124 | 2.818 |
| Pseudostem  (250mg/kg) | Pseudostem  (500mg/kg) | | peels (250mg/kg) | | peels(500mg/kg) |
| 64.1 | 63.8 | | 64.29 | | 70.9 |
| 76.5 | 59.9 | | 60 | | 61.4 |
| 66.1 | 67.4 | | 59.2 | | 58.3 |
| 56 | 74.1 | | 57.15 | | 52.9 |
| 86.1 | 66.8 | | 62 | | 45.2 |
| 74.5 | 64.8 | | 62.3 | | 50.5 |
| 60.8 | 58.9 | | 56.59 | | 55.2 |
| 78.2 | 54.1 | | 63 | | 59.8 |
| **Mean**  70.2875 | **Mean**  63.725 | | **Mean**  60.56625 | | **Mean**  56.775 |
| **Std. Deviation**  10.13 | **Std. Deviation** 6.122 | | **Std. Deviation** 2.791 | | **Std. Deviation** 7.776 |
| **Std. Error of mean**  3.583 | **Std. Error of mean** 2.164 | | **Std. Error of mean** 0.9867 | | **Std. Error of mean**  2.749 |
